# Adaptive responses to fractionated feeding: transient hyperphagia and reversible hepatic lipid deposition in mule ducks

**DOI:** 10.1016/j.psj.2026.107790

**Published:** 2026-09-16

**Authors:** Laura-Lou Zwick, Romain Szczepaniak, Kevin Coustaut, Tsogzolmaa Galbadrakh, Marie Lasserre, Yan Fericelli, Karine Ricaud, Sandra Biasutti, Karine Gontier, Jérôme Roy, Stéphane Panserat, Marianne Houssier

**Affiliations:** a Université de Pau et des Pays de L’Adour, INRAE, NUMEA, Mont de Marsan, 40000, France; b AVIPOLE, INRAE, Benquet, 40280, France

**Keywords:** Duck nutrition, Fractionated feeding, Feeding behavior, Hepatic metabolism, Lipogenesis

## Abstract

This study examines a fractionated feeding protocol and analyzes its effects on feed intake and hepatic energy storage in mule ducks. The protocol was applied to 140 animals and included a 5-day acclimation phase with time-restricted feeding (1 h without quantitative restriction), followed by 21 one-hour meals delivered every 12 h, characterized as the fattening phase. Animals were then returned to *ad libitum* continuous access to food (24 h/day). During the acclimation phase, feed intake increased from 154.97 ± 23.31 to 316.04 ± 22.72 g/animal/meal, reaching 584.59 ± 47.16 g per animal on the first day of fattening phase. Intake remained higher than under *ad libitum* feeding during the first four days of the fattening phase before stabilizing at around 300 g/day/animal. Different measure and sampling were performed before the first meal of the fattening phase, 6 h after the third meal, and after each odd-numbered meal up to the 21st, as well as after the 4-day return to *ad libitum* feeding. Mean liver weight was 66.6 ± 10.6 g before the first meal, increased to 134.0 ± 25.2 g after the third meal, and then decreased thereafter to reach 97.4 ± 18.6 g after the 21th meal. However, liver weight remained higher than under fasting and *ad libitum* feeding conditions for most of the fattening period. Hepatic glycogen remained high (10-15 %) between the third and 21st meals, corresponding to the maximum level achievable by the liver and higher than under continuous *ad libitum* feeding. Total hepatic lipids increased from 5.54 ± 0.542% before the first meal to 7.68 ± 1.55% after the third meal, before decreasing from the fifth meal onward and gradually returning to their initial level over the entire fattening period. Transcript levels were higher after the third meal than those measured prior to the first meal and under *ad libitum* feeding conditions for *ACC, FASN* and *ELOVL6*. Subsequently, expression levels declined to intermediate values, paralleling the decrease in total lipid content. Overall, although lipid accumulation remained limited, the protocol maintained near-maximal hepatic glycogen levels, a key prerequisite for fattening.

## Introduction

In the waterfowl production sector, *foie gras* is mainly obtained from mule duck *(Anas platyrhynchos × Cairina moschata)*, an interspecific hybrid whose metabolic traits favor hepatic fattening. This capacity is exploited through force-feeding, which involves the constrained ingestion of large amounts of feed twice daily for 10 to 11 days, typically resulting in livers weighing between 500 and 700 g, with a total lipid content close to 60% (Lo et al., 2020; Mozduri et al., 2021). The large quantity of feed ingested, often maize rich in starch, initially allows energy to be stored as glycogen, leading to an increase in hepatic glycogen, reaching up to 10-15 % of liver weight. Once glycogen levels are saturated, energy is subsequently stored as lipids (Bonnefont et al., 2019; Mozduri et al., 2021). Although force-feeding efficiently induces hepatic fattening, this practice raises questions regarding animal welfare, notably by restricting mobility, reducing access to environmental enrichment, and imposing repeated forced feed intake (Rochlitz and Broom, 2017).

In this context, growing interest has emerged in the development of alternative feeding strategies designed to reduce reliance on force-feeding while preserving effective hepatic fattening. These approaches aim to stimulate spontaneous hyperphagia, whereby animals voluntarily consume large amounts of feed without the need for forced administration.

Research on spontaneous liver fattening in waterfowl has investigated multiple approaches as alternatives to force-feeding. These include genetic predispositions, with certain species or individuals exhibiting an inherently higher capacity to develop behaviors that promote hepatic fattening; for example, the Greylag goose (*Anser anser*) demonstrates a particularly pronounced propensity for hyperphagic behavior, which facilitates spontaneous hepatic lipid accumulation (Wang et al., 2019; Wei et al., 2021). In addition, environmental factors such as photoperiod (Wang et al., 2016) and temperature (Liu et al., 2022) have been shown to influence feeding behavior and liver metabolism. The potential role of the gut microbiota in modulating lipid metabolism has also been explored (Knudsen et al., 2021). In this context, feed availability, referring to controlled feeding schedules and rationing as opposed to *ad libitum* conditions, has also been investigated as a potential factor influencing feeding behavior and hepatic metabolism. A protocol involving long periods of feed restriction, both in quantity and frequency, followed by extended *ad libitum* refeeding, aims to induce compensatory hyperphagia. This strategy has proven particularly effective in geese (Guy et al., 2013; Fernandez et al., 2016; Knudsen et al., 2025), whereas in ducks, despite similar conditions, the protocol has so far failed to induce significant liver fattening because the hyperphagic behavior cannot be sustained (Knudsen et al., 2017).

Consequently, alternative strategies specifically designed to modulate feeding behavior in ducks remain to be explored. In this context, fractionated feeding, which involves alternating periods of feed restriction (fasting) and brief refeeding (meals), appears to be a promising approach. This feeding strategy has been shown in mammals and birds to stimulate compensatory intake and induce metabolic adaptations, thereby promoting the activation of energy storage pathways (Azis et al., 2013, 2018; Kliewer et al., 2015; Fernandez et al., 2018).

On this basis, fractionated feeding strategies have recently been investigated in mule ducks as a means to directly modulate feeding behavior (Zwick et al., 2026a; Zwick et al., 2026b. This approach involves alternating periods of fasting and one-hour meals without quantitative restriction. Initially, meals are separated by 23-hour fasting periods over five days, serving as an acclimation phase during which animals progressively develop hyperphagic behavior, defined here as the ingestion in a single meal of an amount of food greater than their usual daily intake. After this acclimation phase, fasting periods are reduced to 12 hours to promote hepatic lipid accumulation. Promising study has shown that after three meals spaced 12 hours apart, liver weight and total lipid content increased, reaching average values of 150 g and 10% respectively (Zwick et al., 2026b), which are comparable to those observed following a third force-feeding meal (Bonnefont et al., 2019). These changes were associated with an increase in the expression of genes involved in lipid synthesis, a decrease in the expression of genes involved in lipid oxidation, a rise in plasma triglycerides (TG), and a decrease in plasma free fatty acids (FFA), similar to the molecular and metabolic modifications observed during a period of force-feeding (Tavernier et al., 2017a, 2018; Pioche et al., 2020; Massimino et al., 2021). These observations support the effectiveness of fractionated feeding in modulating both molecular and metabolic pathways underlying hepatic fattening. However, the fractionated feeding protocol has so far only been evaluated after three meals, and the evolution of hyperphagic behavior and hepatic fattening over a longer fattening period remains unknown. It therefore appears relevant to extend the fractionated feeding protocol over a period similar to that of a conventional force-feeding program, in order to characterize the evolution of hyperphagic behavior and hepatic fattening throughout the fattening period. Such an extension would also allow a direct comparison between fractionated feeding and conventional force-feeding over an equivalent feeding period.

The present study therefore aimed to assess the fractionated feeding protocol over a period equivalent to that of a full force-feeding program. Male mule ducks underwent a five-day acclimation phase, receiving a one-hour meal per day separated by 23 hours of fasting, followed by a fattening phase with 21 meals spaced 12 hours apart. The primary objectives were: (1) to characterize feed intake dynamics and determine whether hyperphagic behavior is sustained, and (2) to evaluate hepatic fattening and energy metabolism by measuring liver weight and energy composition, subcutaneous and abdominal fat depots, liver color, plasma metabolic parameters, as well as gene expression involved in both lipid and glycogen storage. By combining all these measures, the study aims to investigate how fractionated feeding influences feed intake patterns and the physiological and molecular mechanisms underlying hepatic energy storage and lipid metabolism in ducks.

## Material and method

### Animals

A total of 140 mule ducks were obtained at day 1 post-hatch, and were allocated to 8 pens (n = 10-30 per pen)**.** The animals were vaccinated against influenza at 10 and 28 days of age. Ducklings were raised under the standard conditions and had *ad libitum* access to a starter diet (PAG1301, SOAL Haut-Mauco, France, supplemental Table 1) until 4 weeks of age. After 4 weeks, birds were transitioned to *ad libitum* growing diet (PAG1320, SOAL Haut-Mauco, France, supplemental Table 1).

The experimental design is illustrated in Fig. 1. Starting at 59 days of age (8.4 weeks), the birds were subjected to a fractionated feeding protocol using the growing diet (PAG1320, SOAL Haut Mauco, France, supplemental Table 1). This protocol began with a 5-day acclimation period, during which ducks were provided free access to feed without quantitative restriction for 1 h per day, from 7:00 to 8:00 AM (meal A1 to meal M1; acclimation phase). At the end of the one-hour feeding period, the animals had ceased feeding despite the continued availability of excess feed in the feeders, confirming that they had reached satiety. Following the acclimation period, birds were given free access to feed without quantitative restriction for 1 h every 12 h, from 7:00 to 8:00 AM and from 7:00 to 8:00 PM, for a total of 21 fractionated meals (meal M1 to meal M21; fattening phase). Ambient temperature was measured at the start of each meal and decreased from 25.11°C at the beginning to 17.74°C at the end of the experimental period. At the end of the fractionated feeding protocol, remaining birds were returned to *ad libitum* feeding with 24 h access to feed for 5 days. Body weight, liver, subcutaneous fat and abdominal fat were weighed, while liver tissue and plasma samples were collected at the following time points: before meal M1 (T0h.M1, after a 23-h fast); and then 6 h after each odd-numbered meal from the 3rd to the 21st meal for body weight, liver, and plasma measurements (from T6h.M3 to T6h.M21); and finally, after 4 days of *ad libitum* feeding with 24-h access (Adlib). For adipose tissues, these same time points were used except for M11, M13, and M15, for which sampling could not be performed because of technical and logistical constraints (n = 10 per sampling point; 120 animals slaughtered out of the 140 present). Ducks were euthanized by exsanguination following electrical stunning in a water bath. Stunning was applied for 85 seconds at 8 pulses per second, 100 V, and 20 mA per duck, in accordance with European Council Regulation (EC) No. 1099/2009 [Council Regulation (EC) No. 1099/2009 of 24 September 2009 on the protection of animals at the time of killing, with EEA relevance]. The procedure was performed at the Palmiped Farming Systems Facility (AVIPOLE, 2023) and approved by the “Animal Health and Welfare” unit.

**Fig. 1:**
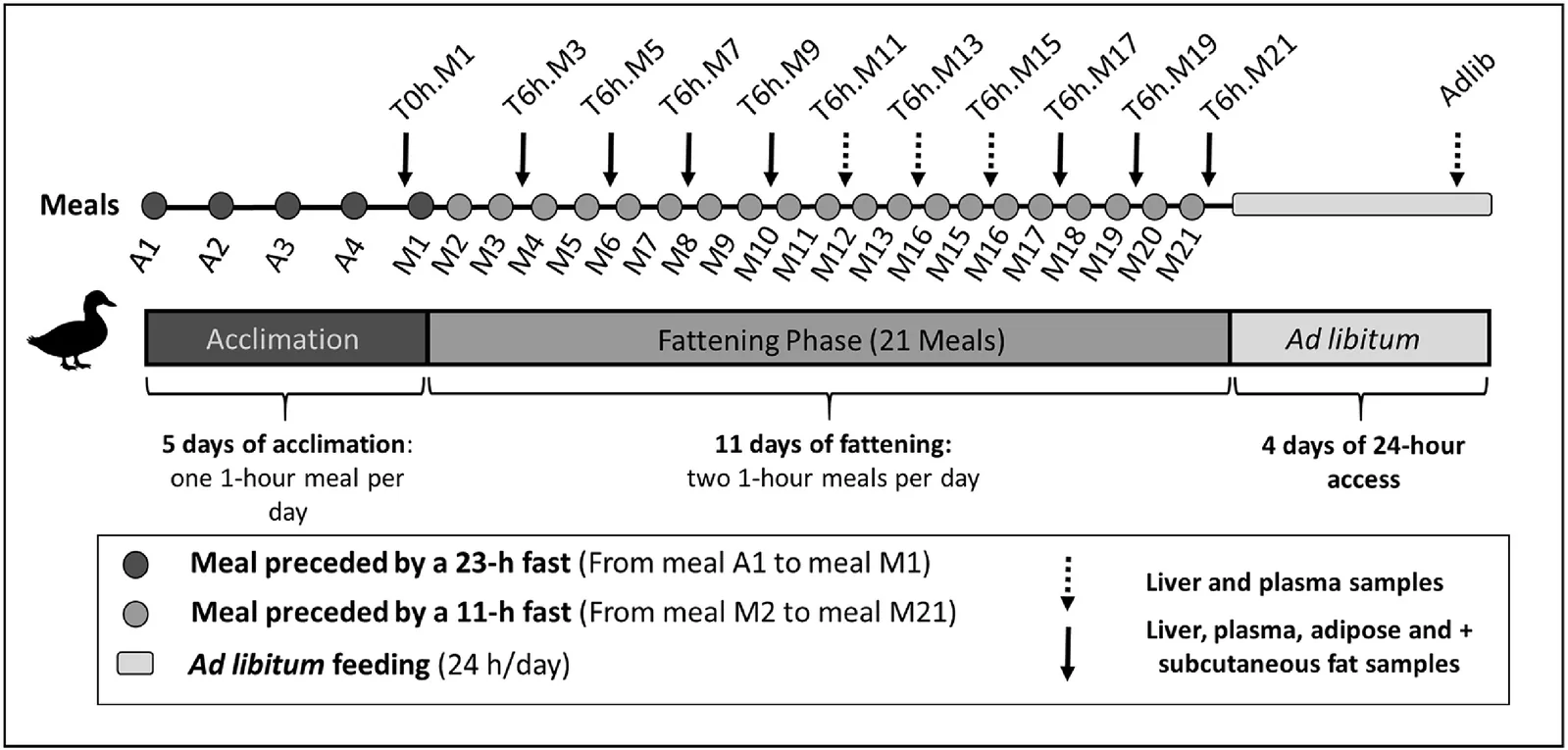
**Experimental design.** A total of 140 mule ducks were subjected to a fractionated feeding protocol. During the acclimation phase, birds received one meal per day, each preceded by a 23.h fasting period (meal A1 - meal M1). This was followed by a fattening phase consisting of 21 sequential meals administered over 11 days. From meal M1 to meal M21, each meal was preceded by an 11h fasting period, thereby implementing the fractionated feeding regimen. Subsequently, ducks were returned to ad libitum feeding with continuous 24h access to feed for 4 days. Tissue and blood samples were collected before the first experimental meal (T0h.M1) and 6 h after selected meals during the fattening phase (T6h.M3, T6h.M5, T6h.M7, T6h.M9, T6h.M11, T6h.M13, T6h.M15, T6h.M17, T6h.M19, and T6h.M21), as well as after the 4.day *ad libitum* refeeding period.

### Sampling and measurements

Feed intake per meal was estimated by subtracting the amount of feed remaining in the feeders at the end of the feeding period from the amount initially provided. The result was then divided by the number of birds with access to the feeder (ranging from 10 to 30 per pen) to calculate the mean feed intake per bird. Total daily feed consumption was determined by summing the intake of all meals consumed within that day (one meal for acclimation phase and two meals for fattening phase). At slaughter, liver color was measured on the large lobe using a colorimeter (Konica Minolta - Color Reader CR-10), recording the CIE L* (lightness), a* (red-green), b* (yellow-blue) values. Liver samples were collected from the central portion of the large lobe for the analysis of lipid and glycogen content, gene expression, and stored at -80°C. Blood was drawn into heparinized tubes and centrifuged at 2,000 × g for 10 min at 4°C to obtain plasma, which was subsequently stored at -20°C until analysis.

### Hepatic energy content

Hepatic lipid and glycogen contents were determined using specific extraction and quantification procedures. For hepatic TG analysis, 100 mg of liver tissue was homogenized in 1 ml of 5% Igepal solution and subjected to two heating cycles at 90°C for 5 minutes each. TG concentrations were then measured with an enzymatic essay kit (Sobioda, Montbonnot-Saint-Martin, Isère, France; product reference: W1W632-50991) according to the manufacturer’s protocol. Total lipids were extracted from 2.5 g of liver tissue using an Ultra-Turrax homogenizer in a dichloromethane/methanol mixture (2:1 v/v) containing 1% butylated hydroxytoluene (Sigma-Aldrich, St. Louis, MO; product reference: B1378) to prevent oxidation. Extracted lipids were quantified using the Folch method (Folch et al., 1957; Cequier-Sánchez et al., 2008), expressed as a percentage of liver weight.

Glycogen content was determined from 150 mg of liver tissue. Free glucose was first measured in homogenized samples with 1 N hydrochloric acid (HCl). Remaining glycogen was subsequently hydrolyzed by heating the samples at 100°C for 2 hours and 30 minutes in HCl. Both free glucose and total glucose (after hydrolysis) were quantified enzymatically (Sobioda, Montbonnot-Saint-Martin, Isère, France; product reference: W1306.140). Hepatic glycogen content was calculated by subtracting the free glucose fraction from total glucose and expressing the result relative to the original 150 mg of liver tissue.

### Gene expressions measurements

Total RNA was extracted from liver samples of adult ducks using TRIzol reagent (Thermo Fisher Scientific, Waltham, MA; product reference: #12034977) following the manufacturer’s instructions. RNA concentration was quantified by measuring absorbance at 260 nm using a Biotek EPOCH 2 microplate reader (Take3 Plate). All RNA samples were subsequently diluted to a uniform concentration of 500 ng/µl for downstream applications.

Reverse transcription was carried out using 2 µg of RNA diluted in 20 µl of RNase-free water. The reaction mixture contained Random Primers (Promega, Madison, WI; product reference: C1181) at a final concentration of 0.2 µg/ml, a PCR nucleotide mix (Promega, Madison, WI; product reference: C1145) at 0.004 mM, and 1 pg of exogenous luciferase RNA at 0.1 pg/µl (final volume of 25 µl). Prior to reverse transcription, RNA samples underwent an initial denaturation step in a CFX384 thermal cycler (BioRad, Hercules, CA) at 65°C for 5 minutes, followed by a 1-minute cooling step at 4°C, after which the RNA was maintained at 4°C. After denaturation, two 12 µL aliquots were taken and designated as RT1 and RT2.

Reverse transcription was then performed to synthesize cDNA from 1 µg of total RNA (final concentration 0.05 µg/µl) using 100 U of SuperScript III Reverse Transcriptase (Invitrogen, Waltham, MA; product reference: 18080044) at 5 U/µl and 40 U of RNaseOUT (Invitrogen, Waltham, MA; product reference: 10777019) at 2 U/µl. The thermal cycler program consisted of 25°C for 5 minutes, 55°C for 1 h, and 70°C for 15 minutes. To verify reaction specificity and rule out contamination, negative controls were included for each group: a RNA-free control (lacking RNA) and a RT-free control (lacking SuperScript enzyme).

Relative gene expression was measured using real-time quantitative PCR (qPCR) with Perfecta SYBR Green FastMix (VWR, Radnor, PA; product reference: 733-1379). Each 5 µl qPCR reaction contained 2 µl of cDNA diluted 1:80, 3 µl of SYBR Green mix, and gene-specific forward and reverse primers at a final concentration of 400 nM (see supplemental Table 2). qPCR was performed on a CFX384 qPCR Detection System (BioRad, Hercules, CA) with the following thermal profile: initial pre-incubation at 95°C for 10 minutes, followed by 45 cycles of 95°C for 15 seconds, 60°C for 10 seconds, and 72°C for 15 seconds. Primer quality was verified prior to qPCR, and melting curve analysis confirmed the amplification of a single specific product for each primer pair. Serial dilution assays were performed to determine primer efficiency, and only primers with efficiency above 85% were included in the analyses. Negative controls, including RNA-free control and RT-free control, were assessed post-amplification to ensure the absence of contamination.

The cDNA corresponding to reference genes, target genes, and exogenous luciferase RNA was amplified for each RT replicate (RT1 and RT2). Exogenous luciferase RNA was added as an external spike-in control to assess the efficiency of the reverse transcription and qPCR reactions and to account for potential technical variability between samples. For reference genes and luciferase, the mean Ct value across all experimental conditions (12 conditions) was checked to ensure that it did not exceed a 1 Ct difference. For reference genes, target genes, and exogenous luciferase RNA, technical replicates (RT1 and RT2) were required to differ by no more than 1 Ct, and only those meeting this threshold were retained. Ct values from accepted duplicates were then averaged for subsequent analysis to ensure reliability and consistency of gene expression measurements.

Relative gene expression was calculated using the 2^-ΔΔCt method as described by Livak and Schmittgen (2001). Normalization was performed using the geometric mean of Ct values from the two most stable reference genes, *STAB1* and *GAPDH*.

$$\text{Relative}\;\text{expression}=\frac{RQ\;target}{RQ\;reference\;\left(geometric\;mean\;of\;the\;Cts\;of\;STAB1\;and\;GAPDH\right)}$$

Gene expression levels were determined using the relative quantification (RQ) method, where RQ *target* and RQ *reference* were calculated as 2^∆Ct^. In this context, ∆Ct represents the deviation of an individual sample’s Ct value from the global mean Ct of all experimental samples. This normalization approach against stable endogenous controls was employed to ensure robust comparative analysis across the entire dataset.

### Plasma metabolic profile

Blood plasma triglyceride (TG), free fatty acid (FFA), and glucose concentrations were determined using commercial enzymatic assay kits, according to the manufacturers’ instructions. TG were measured with the LabAssay™ Triglycerides kit, glucose was quantified using the LabAssay™ Glucose kit and free fatty acids were analyzed with the NEFA-HR2 kit (previously described in hepatic energy content).

### Statistical analysis

All statistical analyses were performed using RStudio (R version R-4.5.3). Data are presented as mean ± standard deviation (SD). The normality of residuals and homogeneity of variances were assessed using the Shapiro-Wilk and Levene tests, respectively. When both assumptions were met, differences among groups were analyzed using a one-way ANOVA followed by Tukey’s post hoc test. When normality was satisfied but homogeneity of variances was not, Welch’s ANOVA was applied, followed by the Games-Howell post hoc test. When normality was not met, data were log-transformed and re-tested. If assumptions were then satisfied, a one-way ANOVA followed by Tukey’s test was performed. Otherwise, a Kruskal-Wallis test followed by Dunn’s post hoc test was used. The Kruskal-Wallis test followed by Dunn’s test was also applied when the sample size in at least one group was lower than six. Statistical significance was considered at *p* < 0.05.

Pearson correlation coefficients were computed using individual-level data for each animal at each sampling time point (fasted state, 11 postprandial time points, and *ad libitum*). Thus, correlations were performed across all individual observations to assess the relationships between gene expression levels and hepatic and plasma parameters. The results were visualized as a heatmap displaying correlations between gene expression and hepatic or plasma parameters, with non-significant correlations (*p* > 0.05) masked.

## Results

### Feed intake

The average feed intake per duck was recorded at each meal throughout the experiment, and the results are presented per meal in Fig. 2.A and per day in Fig. 2.B. The ducks’ feed intake per meal (2.A) changed significantly during the fractionated feeding protocol (*p* < 0.001). During the acclimation phase, with meals spaced 23 h apart, mean feed intake per animal increased from 154.97 ± 23.31 g (A1) to 316.04 ± 22.72 g (M1), corresponding to a 2.04-fold increase. During the fattening phase, intake at the second meal (M2), spaced 12 h from the previous meal, remained stable at 268.55 ± 36.52 g, before decreasing markedly at meal 3 to 57.54 ± 18.42 g, representing a 78.6% reduction and the lowest intake recorded during the experiment. Feed intake subsequently increased at meal 4 to 258.32 ± 36.52 g (M4). Thereafter, feed intake differed between even- and odd-numbered meals, with odd-numbered meals corresponding to the morning feeding and even-numbered meals to the evening feeding. Overall, intake was higher during even-numbered meals, with values ranging from 163.16 ± 30.73 g (lowest value, M10) to 203.51 ± 32.91 g (highest value, M12), whereas odd-numbered meals showed lower intake, with values ranging from 79.31 ± 17.36 g (lowest value, M19) to 140.43 ± 36.76 g (highest value, M7). By comparison, mean feed intake during the four days of *ad libitum* refeeding averaged 211.80 ± 22.62 g and did not differ significantly from that observed during even-numbered meals.

**Fig. 2:**
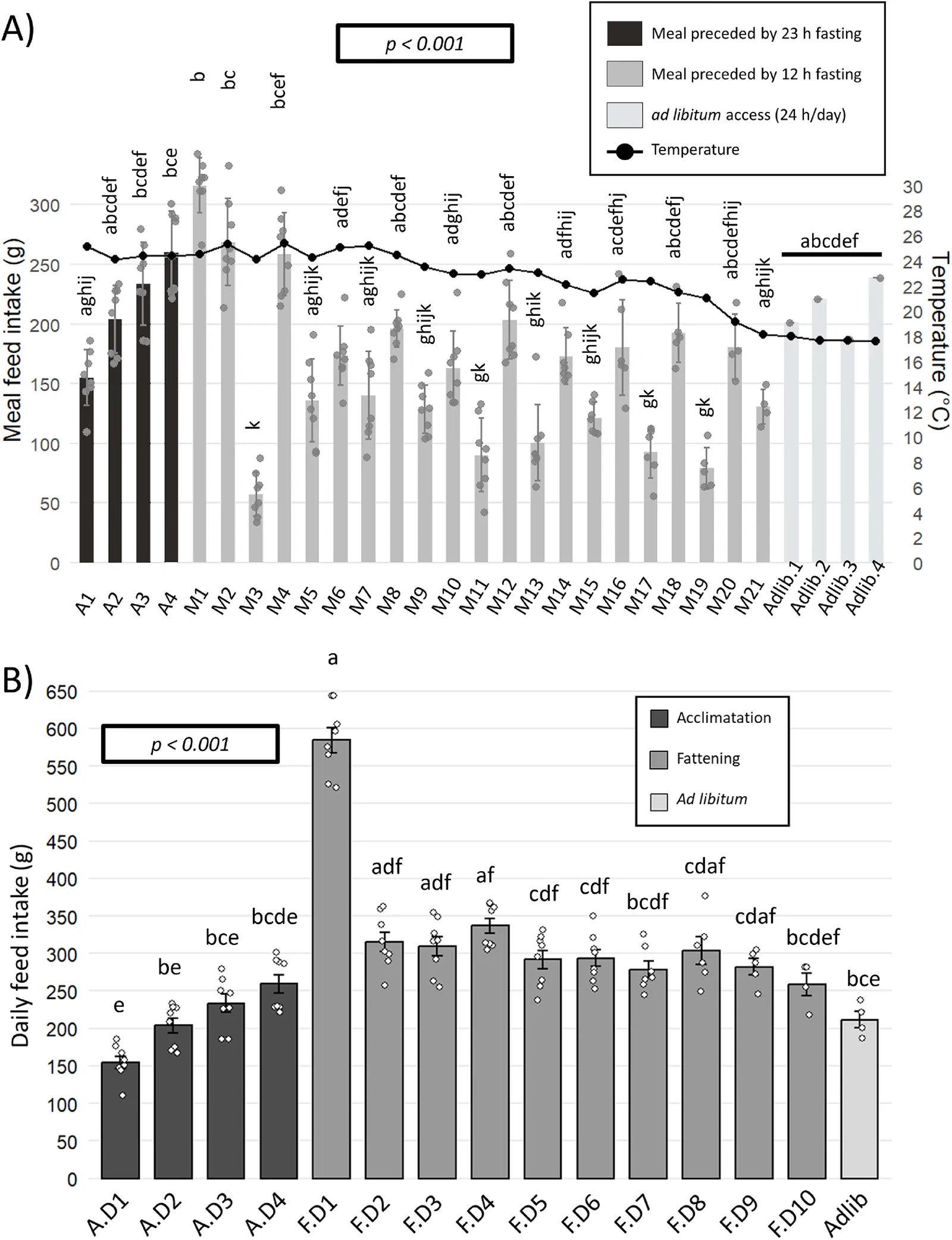
**Feed intake.** Average feed intake per animal was determined per meal (A) and per day (B). Bars represent means ± standard deviation (SD), and dots correspond to average values of each pen (n = 4 to 8 pens). For feed intake per meal (A), Meal.A1 to Meal.M1 correspond to meals preceded by 23 h of fasting (acclimation phase). Meals from Meal M1 to Meal M21 correspond to the 21 meals provided at 12 h intervals (fattening phase). Adlib.1 to Adlib.4 correspond to the 4 days with ad libitum access to feed (24 h/24). Statistical differences in intake between meals were analyzed using a Kruskal-Wallis test followed by Dunn’s post-hoc test. For the *ad libitum* meals with a single measurement, the Kruskal-Wallis test was performed by considering the mean of the four *ad libitum* measurements (Adlib.1 to Adlib.4) as belonging to a single measurement group. Different letters indicate significant differences between times points. Regarding daily feed intake (B), the first 4 days (A.D1 to A.D4) correspond to intake measured during one 1-hour meal per day (acclimation phase). For F.D1 to F.D10, intake corresponds to the sum of the two meals provided daily (fattening phase). The Adlib condition corresponds to the mean intake over the 4 days with 24 h/24 access to feed. Differences in intake between times points were determined using a Kruskal-Wallis test followed by Dunn’s post-hoc test. Different letters indicate statistically significant differences (*p* < 0.05)**.**

Daily feed intake (Fig. 2.B), was significantly altered by the fractionated feeding protocol (*p* < 0.001). Intake increased markedly from 259.46 ± 34.87 g on the last day of the acclimation phase (A.D4) to 584.59 ± 47.16 g on the first day of the fattening phase (F.D1; 2.25-fold increase). Intake remained relatively high until the fourth day of the fattening phase (F.D4), exceeding 300 g per animal, which was higher than intake observed during the first three days of the acclimation phase (one meal per day: maximum 233.54 ± 34.51 g on day three) and higher than the mean intake during the *ad libitum* period (211.80 ± 22.62 g per day).

### Growth and body parameters

Body weight, as well as liver and adipose tissue (subcutaneous and abdominal) weights, were measured at the different sampling points, and the results are presented in Fig. 3. The corresponding body indices for each of these parameters were also determined and are presented in Table 1. Significant differences were observed across the different phases of the protocol for body weight (*p* < 0.044), liver weight (*p* < 0.001) and hepatosomatic index (*p* < 0.001), but did not affect abdominal or subcutaneous fat weights (*p* = 0.809 and *p* = 0.403, respectively) or for subcutaneous and abdominal fat indices (*p* = 0.156 and *p* = 0.982, respectively).

**Fig. 3:**
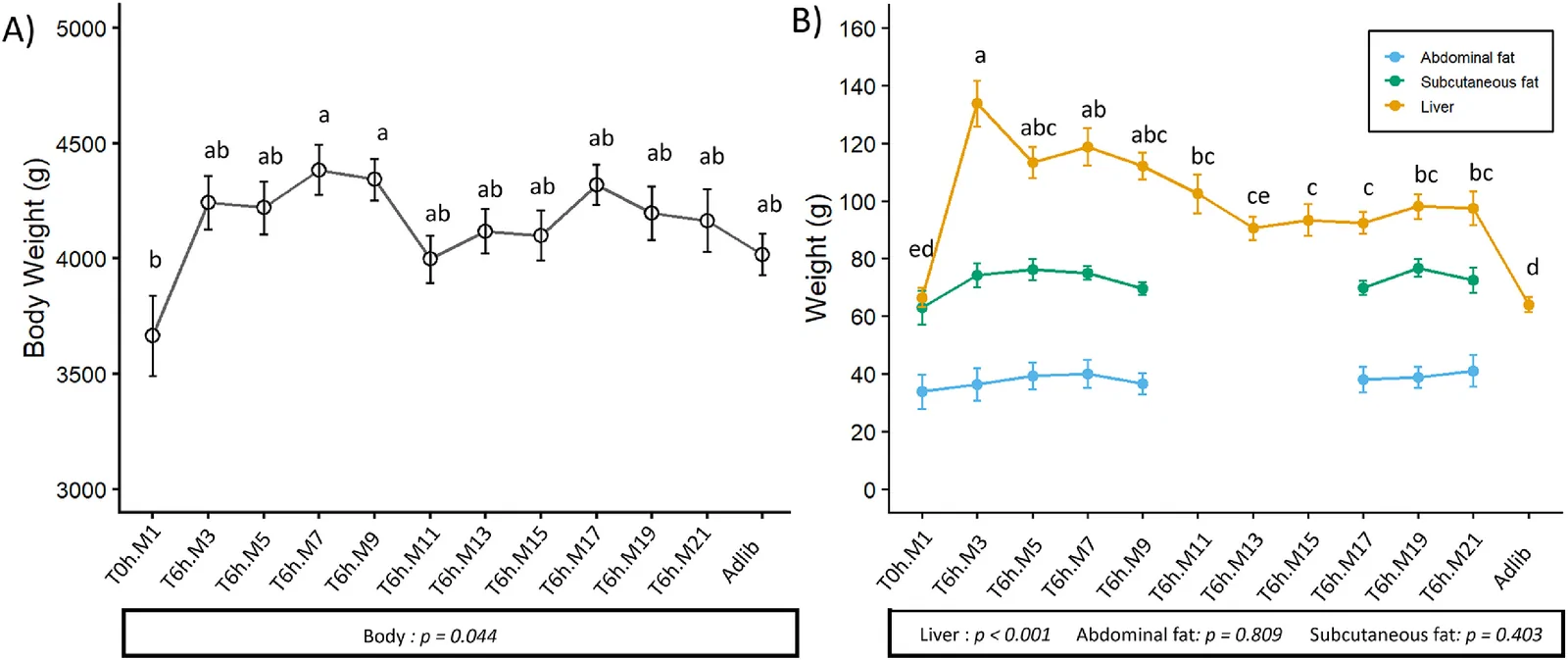
**Morphometric parameters.** Body weight (A) and liver, abdominal, and subcutaneous fat weights (B). Weight changes are presented as line plots showing mean ± standard deviation (SD). Data were analyzed using a Kruskal-Wallis test for body weight, one-way ANOVA for liver weight, and one-way ANOVA on log-transformed data for abdominal and subcutaneous fat masses. When a significant effect was detected between the different time points, an appropriate post hoc test was applied. Different letters (a, b, c) indicate statistically significant differences (*p* < 0.05); n = 10 animals per sampling point.

**Table 1:** Liver and fat body indices.

	Body Indices (% BW (Body Weight))		
	Liver (% BW)	Subcutaneous fat (% BW)	Abdominal fat (% BW)
T0h.M1	1.84 ± 0.29^ef^	1.69 ± 0.37	0.867 ± 0.429
T6h.M3	3.14 ± 0.39^a^	1.75 ± 0.22	0.849 ± 0.395
T6h.M5	2.69 ± 0.37^ab^	1.81 ± 0.23	0.925 ± 0.315
T6h.M7	2.71 ± 0.39^ab^	1.71 ± 0.13	0.904 ± 0.314
T6h.M9	2.58 ± 0.309^bcd^	1.60 ± 0.14	0.839 ± 0.24
T6h.M11	2.56 ± 0.48^bcd^	-	-
T6h.M13	2.20 ± 0.29^cdf^	-	-
T6h.M15	2.26 ± 0.30^bcdf^	-	-
T6h.M17	2.14 ± 0.26^cf^	1.62 ± 0.16	0.873 ± 0.291
T6h.M19	2.34 ± 0.26^bcd^	1.83 ± 0.20	0.928 ± 0.258
T6h.M21	2.33 ± 0.30^bcd^	1.73 ± 0.20	0.964 ± 0.327
Ad.lib	1.59 ± 0.14^e^	-	-
*P-value*	< 0.001	0.156	0.982

Data are presented as mean ± SD (n = 10 per group; total n = 120). For liver index, statistical analysis was performed using one-way ANOVA followed by Tukey’s post hoc test. For subcutaneous and abdominal fat indices, differences between groups were assessed using a Kruskal-Wallis test followed by Dunn’s post hoc test. Different letters indicate significant differences between groups (*p* < 0.05). Missing values indicate that fat measurements were not available for T6h.M11, T6h.M13, T6h.M15, and Ad.lib.

Regarding body weight (3.A), only T0h.M1 (3665 ± 549 g), corresponding to the end of a 23-h fasting period, differed significantly from T6h.M7 (4385 ± 347 g) and T6h.M9 (4342 ± 285 g).

For liver weight (3.B), an increase was observed, from 66.6 ± 10.6 g after a 23-hour fast (T0h.M1) to 134 ± 25.2 g after the third fattening meal (T6h.M3), representing an approximately 2-fold increase. Liver weight remained higher than that observed after the 23-hour fast (T0h.M1) between the third (T6h.M3) and the eleventh (T6h.M11) fattening meals (from 134 ± 25.2 g to 102 ± 21.0 g), before decreasing at the thirteenth meal (T6h.M13) to reach a level comparable to that observed after fasting (90.6 ± 12.9 g), and then stabilizing at an intermediate level until the twenty-first meal (T6h.M21). After four days of *ad libitum* refeeding, liver weight reached a lower value (64.0 ± 7.9 g) than all measurements recorded during the fattening phase, although it did not differ significantly from that observed at the end of a 23-h fasting period (T0h.M1).

The hepatosomatic index followed a similar pattern to liver weight, with significant differences observed across the different phases of the protocol (*p* < 0.001). It increased from 1.84 ± 0.29% BW at T0h.M1, reaching its highest values between T6h.M3 and T6h.M7 (3.14 ± 0.39 and 2.71 ± 0.39% BW, respectively), before decreasing overall during the remainder of the fattening phase. The hepatosomatic index returned to values comparable to those observed at T0h.M1 at T6h.M13, T6h.M15, and T6h.M17, but remained significantly higher than that of the Ad.lib group throughout the entire fattening phase.

### Liver color

The results for liver color are presented in Table 2. Significant changes were observed across the different stages of the protocol for L (*p* < 0.001), a* (*p* < 0.001), and b* (*p* = 0.038). For the lightness parameter (L*), values increased significantly during the fattening period. Specifically, measurements obtained at T6h.M3 (37.6 ± 3.59), T6h.M7 (38.5 ± 3.79), T6h.M13 (44.5 ± 4.57), and T6h.M17 (46.5 ± 5.79) were significantly higher than both the pre-feeding value (T0h.M1; 30.7 ± 1.78) and the *ad libitum* condition (Ad.lib; 32.0 ± 3.0). T6h.M13 and T6h.M17 showed increases of 44.95% and 51.57%, respectively, compared with the value observed before the first meal. T6h.M11 (36.3 ± 4.10) was higher than T0h.M1, but did not differ from the *ad libitum* condition (Ad.lib). The red - green component (a*) followed a different pattern. Only one time point was higher than T0h.M1 (12.1 ± 0.78), namely T6h.M3 (15.7 ± 2.67), although it did not differ from the *ad libitum* condition (Ad.lib ; 12.6 ± 1.70). In contrast, T6h.M17 (6.81 ± 2.97) was lower than both T0h.M1 and Ad.lib, as well as most fattening-phase time points, except for T6h.M7 (11.4 ± 2.27), T6h.M9 (11.1 ± 2.27), and T6h.M13 (8.93 ± 3.16). The yellow-blue component (b*) ranged from 5.1 ± 1.43 at T0h.M1 to 8.24 ± 3.18 at T6h-M1. Although ANOVA indicated a significant effect of protocol stage on b* *(p* = 0.0385), post-hoc comparisons did not reveal any significant pairwise differences.

**Table 2:** Liver color (L, a*, b*) according to the sampling points.

	Color		
	L	a*	b*
T0h.M1	30.7 ± 1.78^e^	12.1 ± 0.783^abcd^	5.1 ± 1.43^a^
T6h.M3	37.6 ± 3.59^c^	15.7 ± 2.67^e^	7.79 ± 2.24^a^
T6h.M5	35.3 ± 2.38^cde^	15.1 ± 2.79^ae^	6.56 ± 1.82^a^
T6h.M7	38.5 ± 3.79^bc^	11.4 ± 2.27^bcdf^	5.71 ± 3.18^a^
T6h.M9	35.4 ± 6.18^cde^	11.1 ± 1.42^bdf^	5.82 ± 2.04^a^
T6h.M11	36.3 ± 4.10^cd^	13 ± 0.76^acde^	8.24 ± 3.18^a^
T6h.M13	44.5 ± 4.57^ab^	8.93 ± 3.16^bf^	6.54 ± 2.08^a^
T6h.M15	35.5 ± 3.07^cde^	12.9 ± 2.02^acde^	6.67 ± 2.11^a^
T6h.M17	46.5± 5.79^a^	6.81 ± 2.97^f^	5.37 ± 1.11^a^
T6h.M19	34.2 ± 2.80^cde^	13.5 ± 1.41^ace^	7.51 ± 2.24^a^
T6h.M21	34.6 ± 2.44^cde^	12.4 ± 1.73^abcd^	6.77 ± 2.57^a^
Ad.lib	32.0 ± 3.0^de^	12.6 ± 1.70^abcde^	5.64 ± 1.94^a^
*p-value*	*p* <0.001	*p* < 0.001	*p* = 0.0385

L*, a*, and b* correspond to the measured liver color parameters: L represents lightness, a* indicates the position on the green - red axis, and b* indicates the position on the blue - yellow axis. Data are presented as mean ± SD (n = 10 per group; total n = 120). For L and b*, statistical analyses were performed using ANOVA on log-transformed data followed by Tukey’s post hoc test. For a*, differences between groups were assessed using a Kruskal-Wallis test followed by Dunn’s post hoc test. Different letters indicate significant differences between time points (*p* < 0.05).

### Liver energy content

Hepatic energy contents in glycogen, TG, and total lipids were also determined and are shown in Fig. 4. All three parameters were significantly influenced by successive feedings (*p* < 0.001 for each parameter).

**Fig. 4:**
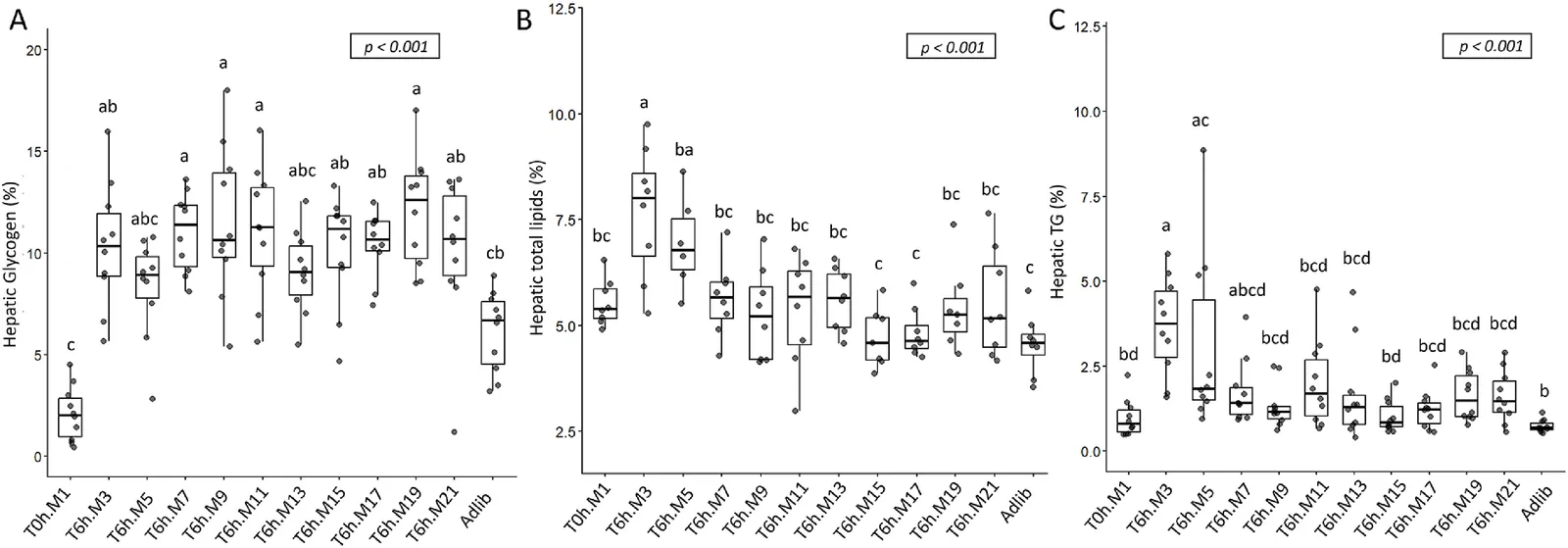
**Energy composition of the liver.** Percentages of glycogen (A), total lipids (B), and hepatic TG (C) are presented as boxplots, with black dots representing individual values. Differences among sampling points were analyzed using a Kruskal-Wallis test followed by Dunn’s post hoc test for glycogen, a one-way ANOVA followed by Tukey’s post hoc test on raw data for total lipids, and a one-way ANOVA followed by Tukey’s post hoc test after log transformation of the data for triglycerides. Different letters (a, b, c, d) indicate significant differences among sampling points (*p* < 0.05). Sample sizes ranged from 6 to 10 animals per sampling point.

Regarding hepatic glycogen (4.A), values measured at the end of the 23-hour fasting period (T0h.M1) were significantly lower than those observed at most other time points, except for T6h.M13 and the *ad libitum* condition, with a mean of 2.11 ± 1.34%. Between this fasting point and T6h.M3, hepatic glycogen increased 4.88-fold, reaching 10.3 ± 3.08%. Subsequently, glycogen levels remained stable at around 10% throughout the fattening phase, with a minimum of 9.07 ± 2.03% (T6h.M13) and a maximum of 12.1 ± 2.77% (T6h.M19). Upon return to *ad libitum* feeding (Adlib), hepatic glycogen levels reached 6.14 ± 1.98%, which was significantly lower than at time points T6h.M7, T6h.M9, T6h.M11, and T6h.M19.

Then, total lipid (4.B) content increased from 5.54 ± 0.54% before the first meal (T0h.M1) to 7.68 ± 1.55% after the third meal (T6h.M3), before progressively declining from meal 5 to meal 17 and then remained at baseline levels throughout the remainder of the fattening phase and after the restoration of *ad libitum* feeding. A similar pattern was observed for TG (4.C), with levels increasing between T0h.M1 and T6h.M3 from 0.977 ± 0.55% to 3.69 ± 1.44%, corresponding to an approximately 3.8-fold increase. TG levels remained elevated until T6h.M5 (3.06 ± 2.57%) before returning to values comparable to those observed after 23 hours of fasting and after a return to *ad libitum* feeding, while showing some fluctuations throughout the remainder of the fattening phase.

### Hepatic gene expressions

The expression levels of liver genes involved in energy metabolism were determined for each sampling point. Genes involved in carbohydrate metabolism and transport, including glycolysis, glycogen synthesis and degradation, as well as glucose transport pathways, are shown in Fig. 5. Regarding genes related to glycolysis, *ENO1*, and *PDHA1* differed significantly between sampling points (*p* < 0.001 for both), whereas *HK1* expression was not affected (*p* = 0.091). The peak expression of *ENO1* was observed after the third meal (T6h.M3) and was higher compared to all points of the fattening phase, except for the fifth meal (T6h.M5). For *PDHA1*, expression increased from before the first meal (T0h.M1) to after the third meal (T6h.M3). This elevated expression was maintained throughout the fractional feeding protocol (fattening phase) and persisted following the return to *ad libitum* feeding (Adlib). Genes involved in glycogen synthesis, *GYS2* and *GBE1*, were also affected during the experimental protocol (*p* < 0.001 for both). For *GYS2*, fluctuations in expression were observed, with T6h.M3 showing higher mean expression than T6h.M7, T6h.M9, T6h.M11, T6h.M13, and the *ad libitum* condition. In contrast, T6h.M7 exhibited lower expression compared with the first three time points (T0h.M1, T6h.M3, and T6h.M5) as well as the last five meals of the fattening phase (T6h.M13, T6h.M15, T6h.M17, T6h.M19, and T6h.M21). Regarding *GBE1*, only the mean expression measured at T6h.M5 was significantly lower than those observed at T6h.M7, T6h.M11, T6h.M13, T6h.M15 and T6h.M17, whereas the expression at T6h.M15 was higher than those at T0h.M1 and T6h.M5. All other conditions, show intermediate levels. Regarding *PYGL*, which is involved in glycogen degradation, no significant differences were observed (*p* = 0.103).

**Fig. 5:**
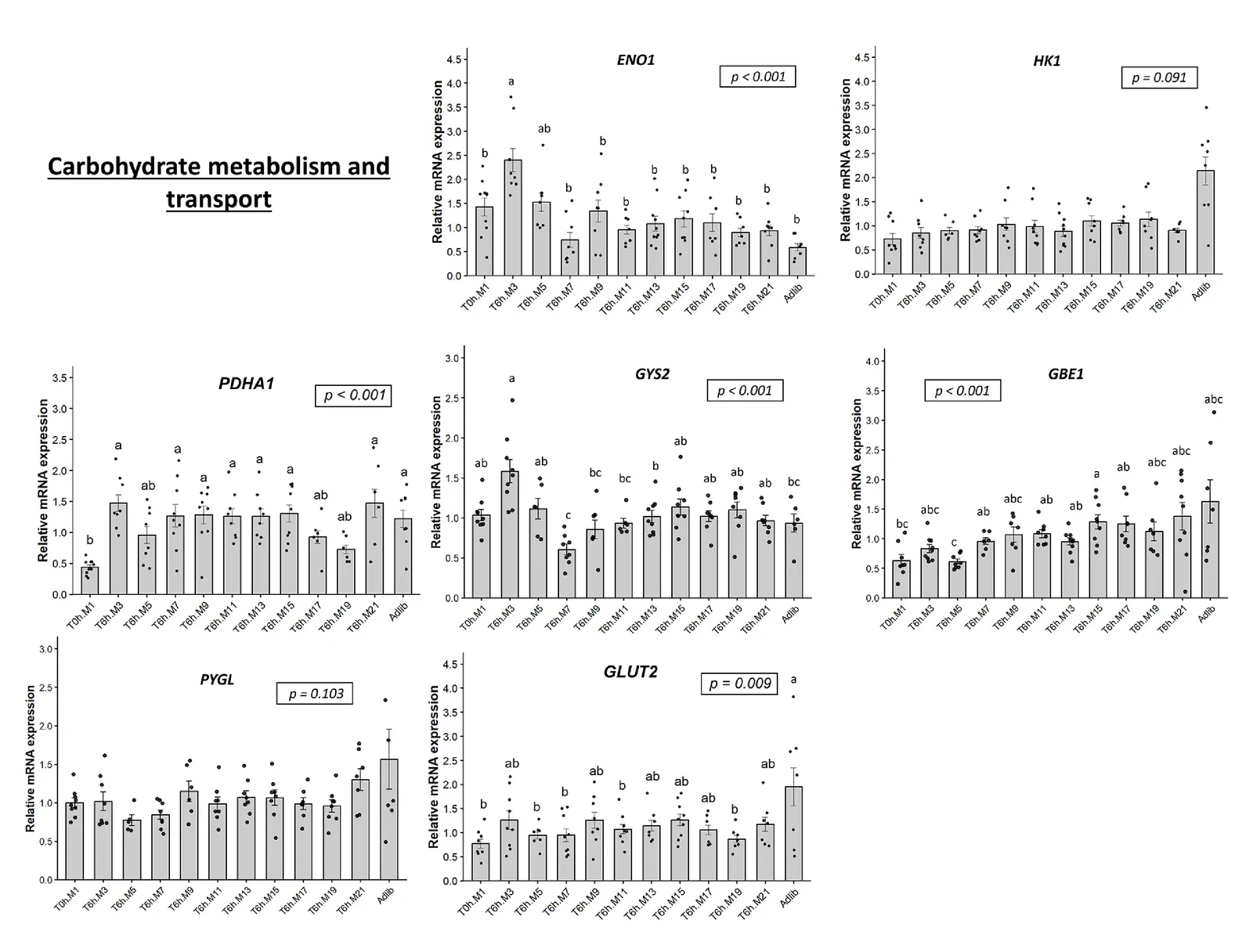
**Relative expression of genes involved in glycolysis, glucose transport, and glycogen metabolism in ducks according to nutritional status.** Expression levels of *ENO1, PDHA1, HK1, GYS2, GBE1, PYGL, and GLUT2* are presented as bar plots showing the mean ± SD, with black dots representing individual animal values. *ENO1, HK1, GBE1*, and *PYGL* were analyzed using Welch’s ANOVA followed by the Games-Howell post hoc test; *PDHA1* and *GLUT2* using one-way ANOVA followed by Tukey’s post hoc test on raw data. Different letters (a, b, c) indicate significant differences among sampling points (*p* < 0.05). Sample sizes ranged from 5 to 10 animals per sampling point.

Finally, regarding *GLUT2*, which is involved in carbohydrate transport, gene expression was also affected by successive feed intakes (*p* = 0.009). The peak expression was observed after the animals returned to *ad libitum* feeding (Adlib). This level was higher than the time points T0h.M1, T6h.M5, T6h.M7, T6h.M11, and T6h.M19, whereas no significant differences were observed at the other sampling points.

The expression levels of genes involved in lipid synthesis were determined and are shown in Fig. 6. The relative expressions of *ACLY, ACC, FASN, SCD1*, and *ELOVL6* were significantly influenced by successive feed intakes (*p* = 0.011 for *ACLY* and *p* < 0.001 for *ACC, FASN, SCD1, ELOVL6*). All of these genes exhibited an initial increase in expression between before the first meal (T0h.M1) and after the third meal (T6h.M3). For ACLY, T6h.M3 was also higher than T6h.M13 and T6h.M17. All other time points during the fattening phase, as well as the *ad libitum* condition, showed intermediate levels and did not differ from any other point. For *ACC*, T6h.M3 was higher than T6h.M7 and the *ad libitum* condition. All other time points showed intermediate values, with only T6h.M11 and T6h.M21 remaining higher than T0h.M1. For *FASN*, T6h.M3 was also higher than the *ad libitum* condition. The remaining fattening-phase time points showed intermediate values and did not differ from either group. For *SCD1*, T6h.M7, T6h.M9, T6h.M19, and T6h.M21 also exhibited higher values than T0h.M1. The remaining time points, including the *ad libitum* condition, displayed intermediate levels and did not differ from one another. For *ELOVL6*, T6h.M3 was higher than all other conditions except T6h.M5, T6h.M19, and T6h.M21. In addition, T6h.M5 remained significantly higher than T0h.M1, T6h.M7, and the *ad libitum* condition. All other time points during the fattening phase showed intermediate values and did not differ from either T0h.M1 or the a*d libitum* condition. Finally, the expression levels of the *GPAT1* and *CEPT1* genes were not affected at all (*p* = 0.172 and *p* = 0.211, respectively).

**Fig. 6:**
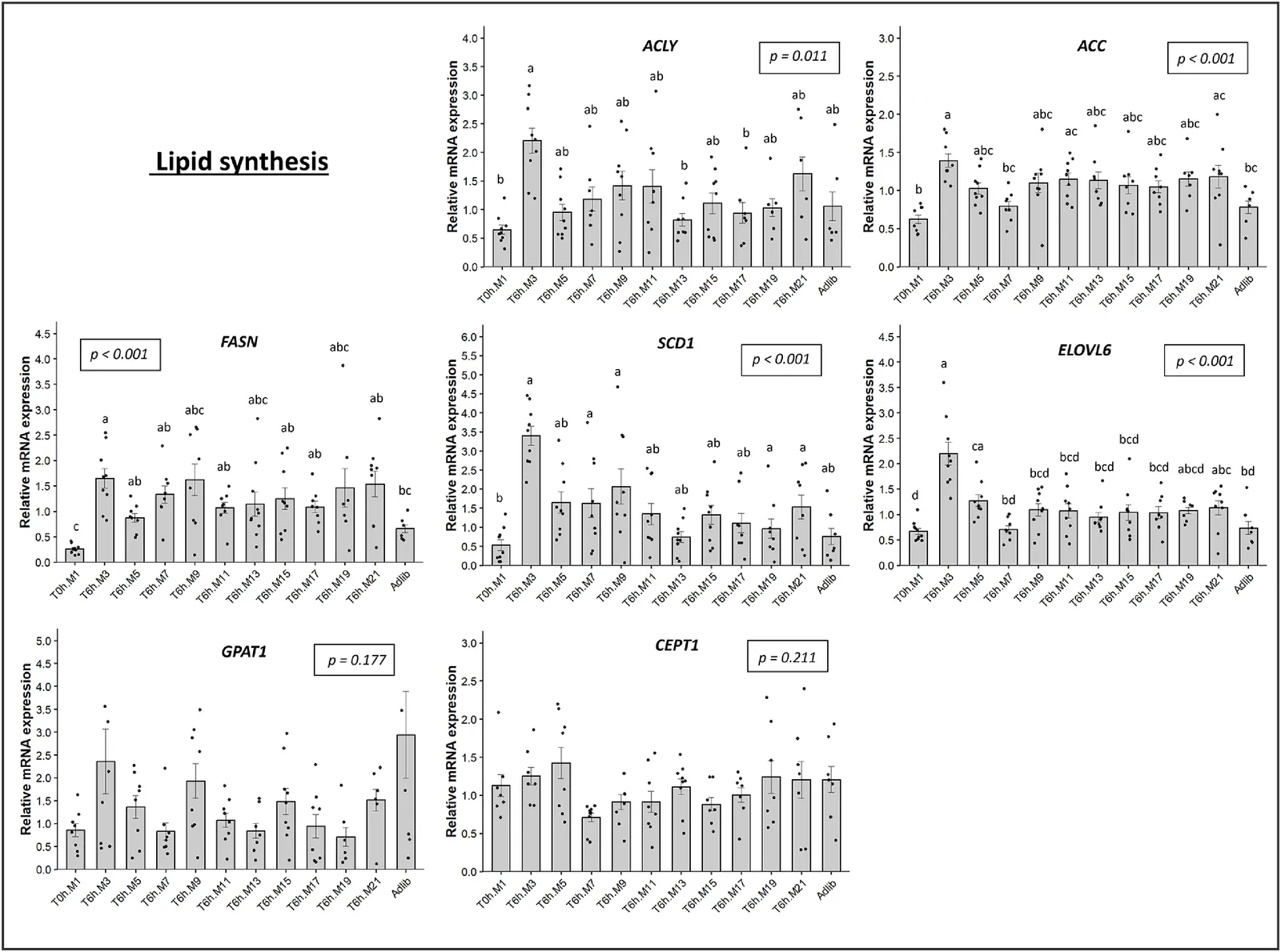
**Relative expression of genes involved in lipid synthesis in ducks according to nutritional status.** Expression levels of *ACLY, ACC, FASN, SCD1, ELOVL6, GPAT1*, and *CEPT1* are shown as bar plots representing the mean ± SD. Black dots indicate individual animal values. Depending on data distribution and homogeneity of variances, statistical differences among sampling points were analyzed using one-way ANOVA for *ACC* and *SCD1* on raw data, one-way ANOVA on log-transformed data for *ACLY* and *GPAT1*, Welch’s ANOVA for *FASN*, and the Kruskal-Wallis test for *ELOVL6* and *CEPT1*. When a significant effect was observed between sampling points, appropriate post hoc multiple-comparison tests were applied. Different letters (a-d) indicate significant differences among sampling points (*p* < 0.05). Sample sizes ranged from 6 to 10 animals per sampling points.

Regarding genes involved in lipid transport (Fig. 7), *LDLR* and *VLDLR* expression levels were not affected by the animals’ successive feed intakes (*p* = 0.051 and *p* = 0.261, respectively). In contrast, *MTTP* expression was significantly modulated (*p* < 0.001), with an increase between the pre-prandial state (T0h.M1) and the postprandial state following the third meal (T6h.M3). Thereafter, expression levels declined from the fifth meal onward (T6h.M5) and remained comparable to baseline values (T0h.M1) for the remainder of the experimental period.

**Fig. 7:**
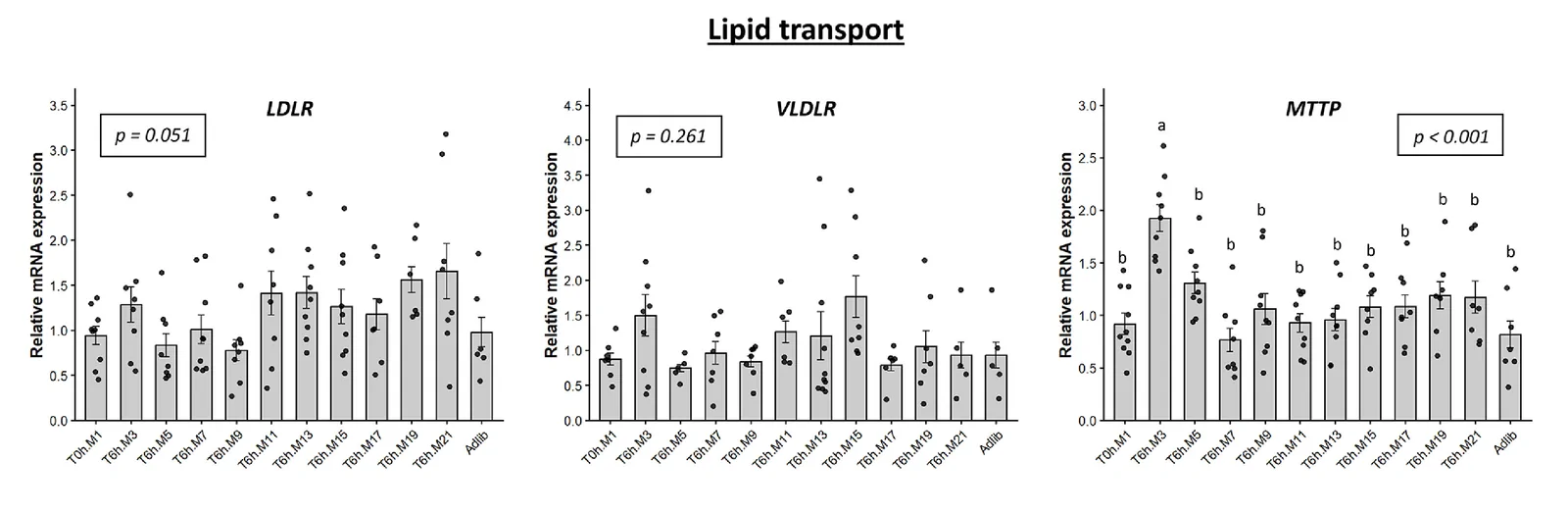
**Relative expression of genes involved in lipid transport in ducks according to nutritional status.** Expression levels of, *LDLR, VLDLR* and *MTTP* are presented as bar plots showing the mean ± SD. Black dots represent individual animal values. Statistical differences among sampling points were analyzed using one-way ANOVA for *MTTP*, one-way ANOVA after log transformation of the data for *LDLR*, and the Kruskal-Wallis test for *VLDLR*. When a significant effect was detected between sampling points, appropriate post hoc multiple-comparison tests were performed. Different letters indicate significant differences among sampling points (*p* < 0.05). Sample sizes ranged from 5 to 10 animals per sampling points.

Finally, regarding genes involved in lipid oxidation (Fig. 8), *ACOX1, ACAD11* and *LIPC* were not affected by successive feed intakes (*p* = 0.118, *p* = 0.678, and *p* = 0.164, respectively), contrary to *CPT1A* and *ACLS1* (*p* < 0.001 and *p* = 0.018, respectively). Regarding *CPT1A* and *ACLS1*, a significant reduction in relative mRNA expression was observed between the pre-first meal time point (T0h.M1) and 6 hours after the seventh meal (T6h.M7). Expression is subsequently maintained to an intermediate level between these two points and remained at this level throughout the remainder of the fractionated-feeding protocol, as well as following the *ad libitum* refeeding phase.

**Fig. 8:**
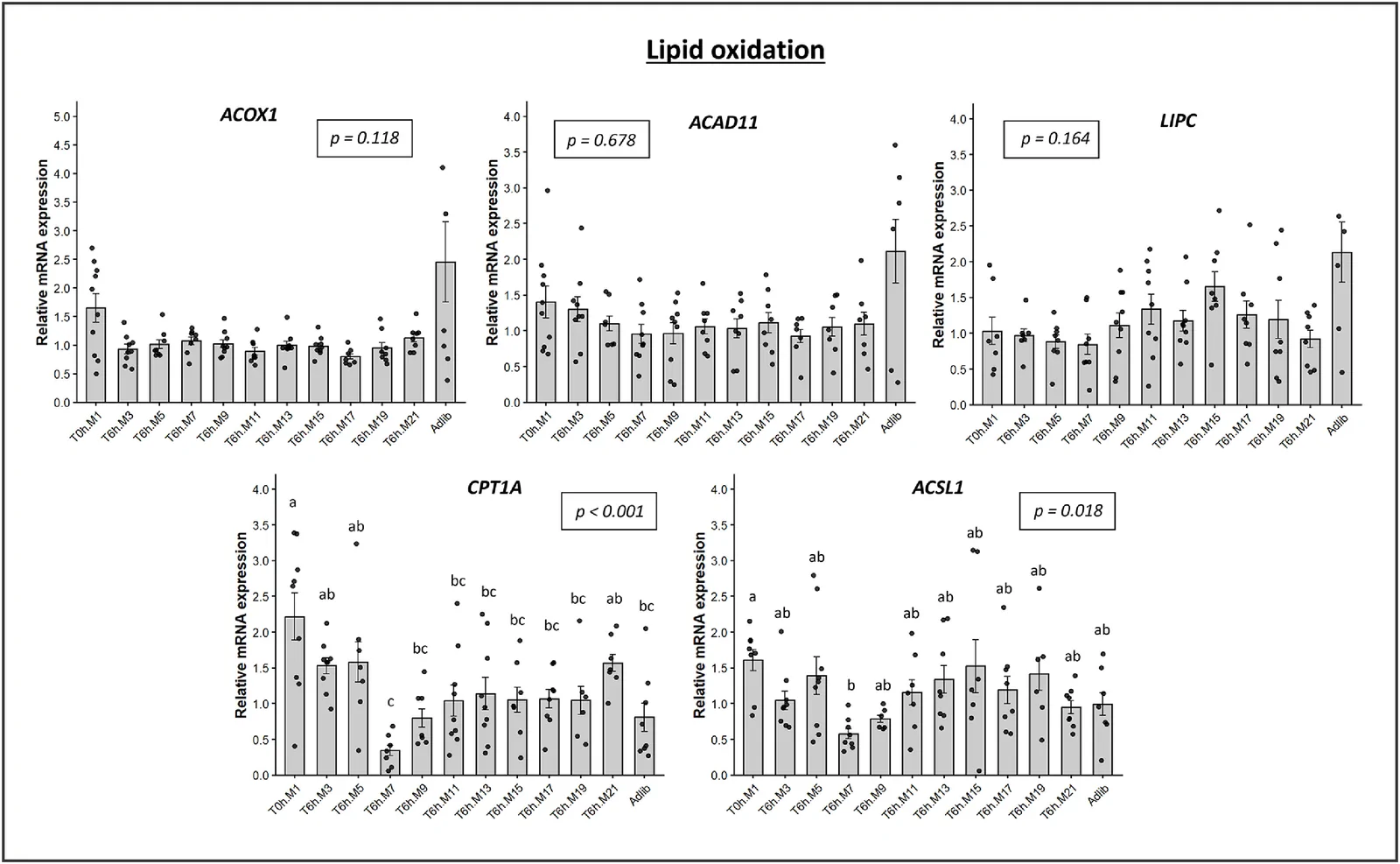
**Relative expression of genes involved in lipid oxidation in ducks according to nutritional status.** Expression levels of *ACOX1, ACAD11, LIPC, CPT1A* and *ACSL1*, are shown as bar plots representing the mean ± SD. Black dots indicate individual animal values. Depending on data distribution and variance homogeneity, statistical differences among sampling points were analyzed using one-way ANOVA (*CPT1A, LIPC*), Welch’s ANOVA (*ACAD11*), or the Kruskal-Wallis test (*ACOX1, ACSL1*). When a significant effect was detected between sampling points, appropriate post hoc multiple-comparison tests were applied. Different letters (a, b, c) indicate significant differences among sampling points (*p* < 0.05). Sample sizes ranged from 6 to 10 animals per sampling points.

### Plasmatic metabolic profile

Plasma metabolite concentrations such as TG, FFA, as well as plasma glucose were determined. The results are presented in Table 3. All parameters were significantly modulated during the experimental protocol (*p* < 0.013 for TG, *p* < 0.001 for FFA, and *p* < 0.018 for glucose).

**Table 3:** Plasma metabolite concentrations (TG, FFA, and glucose) according to the sampling points.

	Plasma metabolites (g/L)		
	TG	FFA	Glucose
T0h.M1	0.94 ± 0.25^b^	0.214 ± 0.061^a^	2.30 ± 0.29^ab^
T6h.M3	1.62 ± 0.67^ab^	0.164 ± 0.060^a^	2.30 ± 0.39^ab^
T6h.M5	1.64 ± 0.72^ab^	0.146 ± 0.041^a^	2.32 ± 0.236^ab^
T6h.M7	1.78 ± 0.74^a^	0.11 ± 0.046^b^	2.19 ± 0.229^b^
T6h.M9	1.46 ± 0.61^ab^	0.14 ± 0.052^ab^	2.26 ± 0.24 ^ab^
T6h.M11	1.39 ± 0.58^ab^	0.11 ± 0.025^b^	2.62 ± 0.26^ab^
T6h.M13	1.29 ± 0.43^ab^	0.167 ± 0.074^a^	2.47 ± 0.369^ab^
T6h.M15	1.50 ± 0.50^ab^	0.13 ± 0.048^b^	2.30 ± 0.39^ab^
T6h.M17	1.39 ± 0.53^ab^	0.101 ± 0.025^b^	2.77 ± 0.389^a^
T6h.M19	1.72 ± 0.76^ab^	0.128 ± 0.033^b^	2.31 ± 0.428^ab^
T6h.M21	1.21 ± 0.35^ab^	0.153 ± 0.054^a^	2.34 ± 0.295^ab^
Ad.lib	1.00 ± 0.29^ab^	0.086 ± 0.035^b^	2.36 ± 0.23^ab^
*p-value*	*p* < 0.013	*p* < 0.001	*p* < 0.018

Plasma triglycerides (TG), free fatty acids (FFA), and glucose concentrations are presented as mean ± SD (n = 9 - 10 per group). For TG and FFA, statistical analyses were performed using ANOVA on log-transformed data followed by Tukey’s post hoc test. For glucose, differences between sampling points were assessed using a Kruskal-Wallis test followed by Dunn’s post hoc test. Different letters indicate significant differences (*p* < 0.05).

Regarding plasma TG, only one significant difference was observed: T0h.M1 showed a lower mean value (0.94 ± 0.25 g/L) compared with T6h.M7 (1.78 ± 0.74 g/L). The other postprandial phases (6 h after meals) showed TG concentrations that remained within a similar range, between 1.21 ± 0.354 g/L and 1.78 ± 0.74 g/L with no significant difference. TG concentrations measured under *ad libitum* conditions did not differ significantly from the other nutritional conditions, with a mean value of 1.00 ± 0.29 g/L.

Regarding FFA, concentrations varied considerably throughout the experimental period, with higher values observed before the first meal (T0h.M1) and at T6h.M3, T6h.M5, T6h.M13, and T6h.M21 (ranged from 0.146 ± 0.041 to 0.214 ± 0.061 g/L) compared with T6h.M7, T6h.M11, T6h.M15, T6h.M17, and T6h.M19 (ranged from 0.101 ± 0.025 to 0.130 ± 0.048 g/L) and the *ad libitum* condition (0.086 ± 0.035 g/L). T6h.M9 showed intermediate values and did not differ from any other time point.

Finally, regarding plasma glucose, only one significant difference was observed between the postprandial point after the seventh meal (T6h.M7; 2.19 ± 0.229 g/L) and that after the seventeenth meal (T6h.M17; 2.77 ± 0.389 g/L). Glucose concentrations remained stable at the other experimental time points, with mean values ranging from 2.26 ± 0.24 g/L (T6h.M9) to 2.77 ± 0.389 g/L (T6h.M17).

### Correlation between gene expression and hepatic and plasma parameters

Then, correlations between hepatic and plasma parameters and the expression of genes involved in hepatic metabolism were analyzed (Fig. 9).

**Fig. 9:**
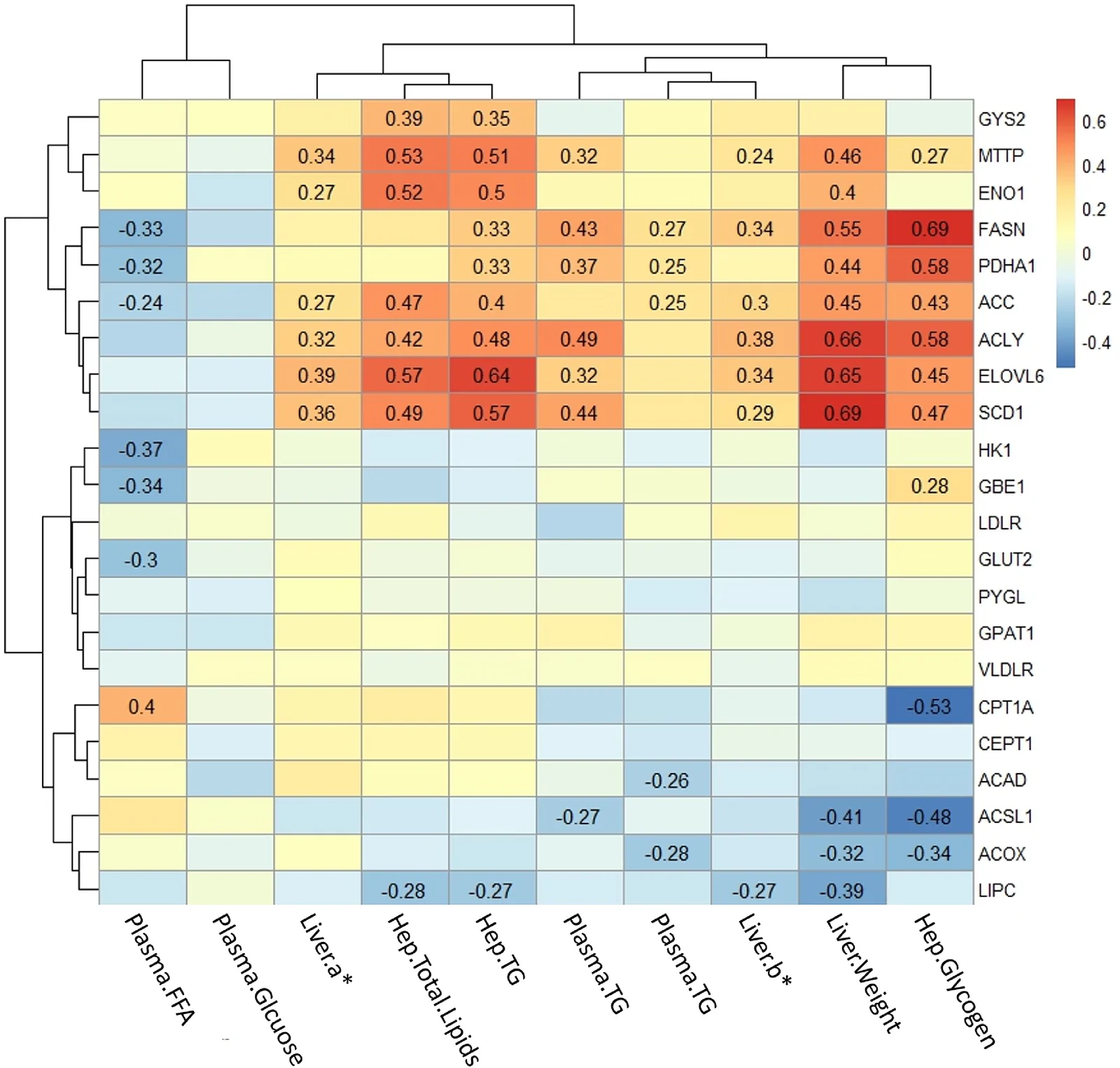
Correlation heatmap between hepatic gene expression and metabolic parameters.

The heatmap represents correlation coefficients between hepatic gene expression levels and metabolic parameters plasma FFA (Plasma.FFA), plasma glucose (Plasma.Glucose), liver weight (Liver.Weight), hepatic glycogen (Hep.Glycogen), total lipids (Hep.Total.Lipids), hepatic triglycerides (Hep.TG), plasma triglycerides (Plasma.TG), and liver color parameters, a* (Liver.a*) b* (Liver.b*), L (Liver.L). Red indicates positive correlations and blue indicates negative correlations, with color intensity proportional to the strength of the correlation (see scale bar). Correlation coefficients are displayed within each cell when significant. Hierarchical clustering was performed on both genes (rows) and metabolic parameters (columns) to highlight similarity patterns.

Regarding physiological parameters, three distinct clusters were identified, reflecting groups of variables showing similar patterns of variation. The first cluster includes plasma FFA, and plasma glucose. The second cluster includes a* parameter for liver color (green - red axis), hepatic total lipids and hepatic TG. The thirds cluster includes liver weight, plasma TG, hepatic glycogen, liver color measured as L* (lightness), and b* (blue - yellow axis).

Regarding genes, three distinct clusters were observed. The first cluster includes genes involved in lipogenesis (*ACLY, ACC, SCD1, ELOVL6, FASN*), glycolysis (*ENO1, PDHA1*), glycogen synthesis (*GYS2*) and lipid transport (*MTTP*), showing significant positive correlations with liver weight (r = 0.4 - 0.69), hepatic TG (r = 0.33 - 0.57), hepatic glycogen (r = 0.27 - 0.69), plasmatic TG (r = 0.22 to 0.32) and liver color (L*, a*, b*) (r = 0.22 - 0.38), while plasma FFA were weakly negatively correlated (r = -0.19 to -0.32). The second cluster includes genes involved in lipid oxidation (*CPT1A*, CEPT1, *ACAD, ACSL1, ACOX, LIPC*), which exhibited weak to moderate negative correlations with liver weight (r = -0.32 to -0.41), hepatic glycogen (r = -0.48 to -0.53), plasma TG (-0.22 to -0.27), and liver color L* (-0.26 to -0.28), while positive correlations were observed with plasma FFA (0.23 - 0.40). Finally, the third cluster comprises genes involved in lipid transport (*LDLR, VLDLR, GPAT1*) and genes involved in hepatic glucose transport and phosphorylation (*GLUT2, HK1*). Few significant correlations were observed for this cluster, except for some weak to moderate negative correlations of *GLUT2* and *HK1* with plasma FFA (r = -0.3 to -0.37) as well as a weak negative correlation between *LDLR* and plasmatic TG.

## Discussion

### Fractionated feeding modulates feed intake dynamics

Previous studies in Pekin ducks have demonstrated substantial inter-individual variability in feed intake (Bley and Bessei, 2008), while feeding behavior can also be influenced by social interactions (Hoppitt and Laland, 2008). In our study, feed intake was measured at the group level, using multiple pens as experimental replicates (4 to 8 depending on the meal), which helped to reduce the impact of inter-individual and social variability on mean feed intake measurements. Although the coefficients of variation between pens ranged from less than 10% to 34.1% across meals, the mean feed intake values and, more importantly, the overall temporal pattern of feed intake were highly consistent with those observed in the previous experiment up to the third meal of the fattening phase (Zwick et al., 2026b). These results suggest that, despite the inherent variability in feeding behavior within groups, the overall feeding response of group-housed mule ducks can be reproducibly modulated by this protocol.

Analysis of average feed intake per meal revealed a marked increase during the acclimation phase. The amount consumed per bird doubled between the first and fifth meal (154.97 g vs. > 300 g), reflecting an adaptation of the animals to the time-restricted access to feed, consistent with previous findings in ducks (Zwick et al., 2026a; b). Moreover, the amounts reached on the last day of the acclimation phase exceeded the daily *ad libitum* intake of 200 - 250 g reported in other studies conducted in Pekin (*Anas platyrhynchos*) and mule ducks (Bley and Bessei, 2008; Basso et al., 2014; Wen et al., 2015), thereby characterizing hyperphagia. This phase involves a gradual learning of the feeding schedule that requires several days for the animals to fully adapt. Behavioral adaptations have been observed in broiler chickens subjected to restricted feeding schedules or light-dark cycles that function as fasting and refeeding protocols, since chickens typically do not feed in darkness. Under these conditions, restricted-fed individuals increase their activity and show heightened motivation around expected feeding times, gradually adjusting their behavior to predictable food availability (May and Lott, 1994; Fondevila et al., 2020). Another hypothesis is that such responses may be associated with morphological adaptations of the digestive tract, particularly in the crop and gizzard as previously observed in chicken and quails (Starck, 1999; Lees et al., 2017; Fondevila et al., 2020), which increase in size to enhance the storage and processing of large meals consumed within a short time frame.

When meal frequency increased from once daily to twice daily, spaced 12 hours apart, the high feed intake was maintained between meal M1 and meal M2, resulting in a daily intake of 584 g on the first day of the fattening phase, which is markedly higher than the daily consumption of a duck under *ad libitum* conditions (Bley and Bessei, 2008; Basso et al., 2014). These observations are in line with what has recently been demonstrated in ducks, where food intake was maintained across the first two meals spaced 12 hours apart following a similar 5-day acclimation period (Zwick et al., 2026b). The high feed intake observed on the first day of fattening phase can be explained by the ducks’ prior habituation to consuming large quantities during a single one-hour daily meal in the acclimation phase. The sudden transition to two meals per day, with a reduced fasting interval of 12 hours, likely induced a surprise effect or a mismatch between the animals’ expectations and the new feeding schedule. As a result, the ducks appear to have maintained a high level of feed intake, comparable to that typically observed during standard force-feeding curves (Bonnefont et al., 2019).

However, this high feed intake was not maintained, as a marked drop was observed at the third meal of the fattening phase (meal M3) with intake decreasing to 57 g, likely reflecting satiety resulting from the two preceding very large meals. The decline in feed intake after three meals spaced 12-hour apart has been previously reported in mule ducks, demonstrating that this response is highly reproducible (Zwick et al., 2026b). A rebound in consumption occurred at the fourth fattening meal (meal M4), reaching a level comparable to that of the second meal (meal M2). En Subsequently, feed intake followed a sawtooth pattern, with higher intake during even-numbered meals (evening) than odd-numbered meals (morning). The observed difference in feed intake between evening and morning meals may generally reflect the combined influence of environmental and temporal factors, such as temperature, light exposure, age, and meal timing, which have been shown to modulate feeding behavior in avian species (Xin et al., 1993; May and Lott, 1994; Noiva et al., 2014; Fondevila et al., 2020; Liu et al., 2022). In our study, these differences do not appear to be related to the age of animals, nor to temperature, which remained stable between morning and evening. Although lighting conditions were similar at the time of the meals, a circadian effect cannot be completely ruled out, as ducks may anticipate day and night, which could contribute to the observed variations in feed intake. In chickens, predictable periods of darkness and feed restriction have been shown to induce anticipatory feeding behavior, characterized by an increase in feed intake before lights-off (May and Lott, 1992). A similar mechanism could partly contribute to the higher feed intake observed during evening meals here. In ducks, feeding activity follows a distinct daily rhythm, with periods of increased feeding activity occurring around the transition between light and dark periods and during the early morning. This suggests that both evening and morning meals may coincide with periods of increased motivation to feed (Bley, 2005; Zhu et al., 2017). Furthermore, the intake pattern may also be explained by the satiety induced on the first day of fattening, with ducks consuming less following a large meal. On the other hand, the higher activity levels generally observed during the daytime (Korner et al., 2016) may increase energy expenditure throughout the day and could therefore contribute, at least in part, to the greater feed intake observed during evening meals. Finally, feed intake at a given meal is likely to be influenced by the amount consumed at the preceding meal. A large meal may temporarily reduce feeding motivation through the activation of satiety signals and the persistence of feed within the digestive tract, thereby limiting intake at the subsequent meal (Tolkamp et al., 2012). This factor could therefore also contribute to the differences in feed intake observed between morning and evening meals. However, despite this variability between morning and evening meals, daily feed intake remained stable for the remainder of the fattening phase, while still being higher than that of *ad libitum*-fed animals during the first four days of this phase, with an increase of approximately 100 g, corresponding to a 50% rise in consumption. This suggests that fractionated feeding allows greater intake compared to *ad libitum* feeding. However, this effect appears to be transient, with intake rapidly self-regulating toward levels similar to those observed under *ad libitum* conditions. These findings are in line with a previous study in geese, where meal-fed birds exhibited elevated feed intake compared with *ad libitum*-fed controls around 8 weeks of age, followed by a subsequent stabilization at intake levels comparable to the *ad libitum* group around 9 weeks of age, thereby confirming the transient nature of this response (Liu et al., 2020). This pattern could reflect a possible adaptive response to fractionated feeding, in which the temporal restriction of feed access might initially stimulate compensatory hyperphagia, leading to higher intake than under *ad libitum* conditions. However, this effect appears to be transient and may be followed by a progressive normalization of intake, suggesting the potential involvement of regulatory mechanisms that could restore the balance between intake and energy requirements over time. These mechanisms may include, among others, adjustments in satiety signaling, which might progressively contribute to limiting feed intake. These results notably confirm the ability of mule ducks to rapidly regulate their feed intake. One hypothesis is that ducks, and more specifically mule ducks, are phylogenetically more distant from their wild migratory ancestor than Greylag geese, which are more closely related from an evolutionary perspective. As a result, they may have largely lost the innate hyperphagic response associated with migratory preparation (Knudsen et al., 2017).

It is worth noting that ambient temperature decreased from 25.11°C to 17.74°C over the course of the protocol, which could have contributed to an increase in feed intake, as previously reported in waterfowl (Liu et al., 2022; Xu et al., 2024). However, given the relatively stable feed intake observed during the fattening phase, any potential contribution of ambient temperature appears to have been limited and was insufficient to promote a substantial increase in feed intake.

However, no meal was completely skipped during the fattening phase, suggesting that feed intake could still be further optimized in future studies, for example by adjusting the type of feed or the timing of meals. Future studies could investigate the kinetics of hepatic energy reserve mobilization following the first two meals, as well as feed transit time through the duck’s digestive tract, in order to optimize the timing of the subsequent meal. Ideally, the next meal would be offered when the animals are sufficiently hungry to maintain a high level of feed intake, while hepatic energy reserves have not yet been substantially mobilized.

### Metabolic adjustments associated with fractionated feeding

***Effects of fractionated feeding on body and fat weights***. Regarding body weight, only the 23-h fasting phase had an impact, resulting in a decrease compared with T6h.M7 and T6h.M9, but without any significant difference compared with the other conditions including *ad libitum*-fed animals. This result could be attributed to a transient effect of short-term fasting on body weight, as previously reported in avian species (Becker et al., 1985; Krause et al., 2017; Katsumata et al., 2024; Liu et al., 2024), notably in ducks (Zwick et al., 2026a). In contrast, the entire fattening phase did not produce any notable difference in body weight compared with birds reintroduced to *ad libitum* feeding. These findings are consistent with previous observations in geese and ducks, where meal-feeding with fasting periods shorter than 12 h did not affect body weight (Liu et al., 2020; Helbawi et al., 2025). Similarly, repeated and more prolonged fasting periods, ranging from 2 to 4 days per week, did not result in body mass loss in male black ducks compared with daily-fed animals (Barboza and Jorde, 2002). Together, these studies highlight the high capacity of ducks to tolerate intermittent feed deprivation, notably through compensatory feed intake, allowing them to maintain their body mass. Moreover, fractionated feeding does not appear to lead to peripheral fat storage, as no differences were observed in the weights or body indices of either subcutaneous and abdominal fat, consistent with what has also been observed in chickens (Fontana et al., 1993).

***Fractionated feeding induces changes in hepatic parameters.*** Regarding liver weight and hepatosomatic index, the observed increase after the third meal suggests that the early meals of the fattening phase contribute to hepatic energy storage and liver mass accretion, likely driven by a cumulative effect of feed intake, as previously reported in ducks (Zwick et al., 2026a; b). Despite subsequent stabilization and decline, liver weight remained higher than under *ad libitum* conditions throughout the fattening phase, indicating a sustained effect of fractionated feeding on hepatic energy storage. Although it involved a different type of fractionated feeding pattern, similar results have been observed in chickens, where a *skip-a-day* feeding program resulted in a higher liver weights or hepato somatic index compared with daily-fed or *ad libitum* birds (De Beer et al., 2008; Lees et al., 2017; Avila et al., 2023).

Fractionated feeding also altered liver color. The liver color is known to indicate the energy content: indeed, a lighter liver with a more yellow hue is generally associated with a higher lipid content (Bonnefont et al., 2019; Fernandez et al., 2019; Abdullah and Buchtová, 2022), but is also associated with increased glycogen levels during the first days of force-feeding (Bonnefont et al., 2019). In the case of the fractionated feeding protocol, the fluctuating increase in L* and a* values during the fattening phase, indicates lighter and transiently redder livers under fractional feeding. Overall, liver color values tended to be higher than those observed under both fasting and *ad libitum* conditions, potentially indicating the establishment of hepatic reserves during the fractional feeding protocol, which may partly explain the increase in liver weight. The variation observed during the fattening phase may indicate fluctuations in hepatic energy content. This effect was accompanied by only a slight and non-significant increase in b*, suggesting a limited impact on the blue - yellow axis. Overall, these changes were less pronounced than those reported under force-feeding condition, where markedly higher color values have been described (L = 46.5 maximum for our protocol versus 65 for force-feeding; b* = 7.79 maximum versus 34 for force-feeding) (Bonnefont et al., 2019).

The establishment of hepatic reserves was notably confirmed by the analysis of liver energy content. First, hepatic glycogen levels were very low after the 23-hour fasting period, indicating near-complete utilization of glycogen reserves to meet energy demands, consistent with previous observations (Zwick et al., 2026a; b). During the fattening phase, glycogen levels remained consistently elevated throughout, with higher values observed at most meals showing higher values than in compared with animals returned to *ad libitum* feeding. This suggests that fractionated feeding helps maintain maximal glycogen stores (approximatively 10% of liver weight). Once these glycogen stores are saturated, excess glucose is redirected toward hepatic *de novo* lipogenesis, leading to promoting fatty acid and TG synthesis (Bonnefont et al., 2019; Mozduri et al., 2021; Zwick et al., 2026b). Hepatic TGs and total lipids peaked between the third and fifth meals, likely due to high initial food intake that rapidly saturated glycogen stores and enabled the synthesis and subsequent accumulation of lipids. These results are consistent with observations in chickens under split-feeding protocols, which promote both substantial hepatic glycogen storage and lipid accumulation in the liver (De Beer et al., 2008; Lees et al., 2017). However, from the second day onwards, feed intake regulation was sufficient to maintain glycogen deposition but inadequate for further lipid accumulation, explaining the observed decrease in lipid proportion. The lighter color observed (higher L value) would therefore most likely be mainly related to glycogen accumulation. This interpretation could be supported by the clustering analysis in the correlation matrix, where Liver L cluster was more closely associated with hepatic glycogen than with total hepatic lipids or hepatic TG. Furthermore, liver weight and hepatic glycogen percentage are grouped within the same cluster, whereas total hepatic lipids and hepatic TG form a more distant cluster. Overall, These patterns suggest that variations in liver weight may be more closely related to hepatic glycogen content than to hepatic lipid accumulation in the present study.

Overall, extending the fattening period to 21 meals administered at 12-hour intervals did not lead to greater hepatic lipid accumulation than that observed after only three meals. This limited response was likely due, at least in part, to the rapid regulation of feed intake, particularly the very low intake observed at the third meal (M3). Indeed, the magnitude and duration of the postprandial increase in liver weight, reflecting hepatic energy storage, depend at least in part on the amount of feed consumed (De Beer et al., 2008; Zwick et al., 2026b). Here, the consumption of a meal averaging less than 60 g likely resulted in rapid utilization of the ingested energy, with limited opportunity for significant energy storage. It may also have promoted the mobilization of previously accumulated energy reserves to meet energy requirements during the 12 h preceding the subsequent meal, as observed during periods of fasting (Scanes and Braun, 2013; Lees et al., 2017). Thus, despite the high feed intake measured at M4 (>250 g), the energy ingested appears to have been primarily directed toward replenishing hepatic glycogen stores, without generating the energy surplus required to promote hepatic lipid accumulation. These results suggest that the third meal may represent a critical point in the protocol. Preventing a sharp decline in feed intake at this stage could help maintain the energy reserves accumulated during the preceding meals, including hepatic lipid stores. Further studies should therefore investigate whether maintaining a higher feed intake at this stage can promote the sustained accumulation of hepatic lipids.

For comparison, force-feeding protocols show an initial increase in hepatic glycogen up to the second day, after which glycogen stores saturate and lipid percentages progressively rise, reaching up to 60% after 10 days, accompanied by a corresponding decline in glycogen proportion (Bonnefont et al., 2019).

### Fractionated feeding induces transcriptional changes in genes involved in liver metabolism

To investigate the mechanisms underlying the observed hepatic alterations, we next assessed the relative expression of genes involved in hepatic energy metabolism, focusing on those previously reported to be activated under force-feeding (Tavernier et al., 2017a, 2018; Massimino et al., 2021) or fractional feeding conditions (Andrieux et al., 2023; Zwick et al., 2026a).

Regarding *GLUT2*, only the *ad libitum* condition showed significantly higher values than those observed both under fasting condition and at certain time points during the fattening phase. The relative stability of its expression throughout the fattening period may be explained by a sampling time that was too late relative to its postprandial expression peak, reported to occur around 2 hours after feeding, rather than 6 hours post-feeding (Tavernier et al., 2017a; Pioche et al., 2020). Under *ad libitum* conditions, more frequent and asynchronous feeding could account for both the observed increase and the high intra-group variability (Bley and Bessei, 2008; Basso et al., 2014). Regarding genes involved in glycogen synthesis and degradation, temporal changes in the expression of *GYS2* (glycogen synthase) and *GBE1* (glycogen branching enzyme) suggest fluctuating transcriptional regulation that appears to be only weakly influenced by nutritional status (fasted versus fed animals), in agreement with several studies in mammals (Doi et al., 2010; He et al., 2022). Finally, *PYGL*, involved in glycogen breakdown, showed no evidence of transcriptional modulation, suggesting that in both ducks and mammals, its regulation is mainly exerted at the post-translational level through phosphorylation and allosteric mechanisms (Agius, 2015).

For genes involved in glycolysis, lipid synthesis, and lipid transport, two distinct patterns of relative expression can be observed: some genes, such as *ENO1, ACLY, ELOVL6,* and *MTTP* exhibit a single peak at T6h.M3, i.e. 18h after the ingestion of the two largest consecutive meals (M1 and M2), reflecting a sustained activation to cope with a massive glucose intake. This pattern is consistent with previous studies showing that these genes markedly respond to large nutrient loads, highlighting their role in coordinating hepatic adaptations to feeding (Tavernier et al., 2017a; Hérault et al., 2019). Other genes, including *PDHA1, ACC, FASN*, and *SCD1*, show intermediate to high levels throughout the fattening phase compared to the fasting phase, suggesting a lower activation threshold and a sustained capacity to maintain hepatic lipogenesis between meals. However, for most genes (with the exception of *ELOVL6* at T6h.M5), expression levels during the fattening phase, except after the third meal, did not differ from those observed after the return to *ad libitum* feeding. These results suggest that fractionated feeding induced an increased expression of these genes only after the third meal, but that this upregulation was not sustained over time. This transient response may explain the return to baseline lipid levels observed after the third meal.

Regarding genes involved in lipid oxidation (*CPTIA* and *ACSL1*), the decrease in expression levels between before the first meal and after the seventh meal suggests a shift from a fasting-induced mobilization state toward a deposition state. This pattern is consistent with the metabolic adaptations commonly observed in ducks, where lipid oxidation is enhanced during fasting and reduced during the postprandial period (Andrieux et al., 2023; Zwick et al., 2026a). These results correspond in particular to what can be observed during a force-feeding period, during which the expression of these genes decreases following a large intake of feed (Tavernier et al., 2018; Pioche et al., 2020; Massimino et al., 2021). Subsequently, the expression levels of these genes return to values comparable to those observed under *ad libitum* conditions, indicating that, even in the presence of a constant energy supply, lipids continue to contribute to energy metabolism.

Overall, these results help explain the absence of further lipid accumulation beyond the third meal during the fattening phase, likely due to the inability to induce lipogenesis above the level observed under *ad libitum* feeding conditions, together with the maintenance of active lipid utilization as an energy source. This may be closely related to feed intake, which was higher during the first two fattening meals (meal M1 and M2) and then decreased. Of course, more in-depth studies of protein levels or associated enzymatic activities would be needed to confirm this hypothesis, but correlation analyses highlighted a coherent organization linking hepatic phenotype to transcriptional regulation. The positive associations observed between anabolic genes and hepatic storage parameters support a coordinated activation of lipogenic and glycolytic pathways, contributing to lipid and glycogen accumulation. Conversely, the negative correlations involving genes related to lipid oxidation suggest that reduced oxidative capacity may promote this storage-oriented phenotype. These findings are consistent with patterns typically observed during hepatic fattening, whether under force-feeding protocols (Tavernier et al., 2017a; b; Massimino et al., 2021) or fractionated feeding protocol (De Beer et al., 2008; Andrieux et al., 2023; Zwick et al., 2026a).

***Changes in blood parameters with fractionated feeding.*** The hepatic observations are supported by plasma changes. Plasma triglycerides increased after feeding, with peak values coinciding with periods of high hepatic lipid content, suggesting efficient hepatic lipid export and contributing to subsequent decreases in liver lipid levels. This pattern is consistent with previous studies in chickens and ducks showing postprandial increases in circulating TGs (Shiau et al., 1985; Tavernier et al., 2018; Andrieux et al., 2023; Wang et al., 2024; Zwick et al., 2026a). Circulating FFAs, reflecting adipose tissue lipolysis, were elevated after the 23-hour fast, indicating increased energy demand and mobilization of lipid reserves, as commonly reported in avian species during fasting (Serr et al., 2009; Honda et al., 2017; Xiao et al., 2020). During the fattening phase, FFA levels significantly decreased after the seventh meal, likely reflecting a preferential glucose utilization following the first large meals. FFA levels subsequently increased before increasing at later time points coinciding with low feed intake (M13), and the end of the protocol (M21), possibly reflecting a gradual depletion of hepatic energy reserves and the mobilization of peripheral energy stores. The low FFA levels observed under *ad libitum* feeding, likely reflects a continuous supply of dietary glucose, which meets energy demands and reduces the need for lipid mobilization from adipose tissue. Concerning glycemia, blood glucose varied little between the fasting phase, 6 hours after meals, and *ad libitum* conditions, which is consistent with previous observations in ducks, whose glycemia is generally maintained within a relatively stable range Concerning plasma glucose, concentrations remained remarkably stable, regardless of feeding status (fasted, 6 h after a meal, or fed *ad libitum*). This finding is consistent with the well-established characteristics of avian glucose homeostasis, whereby blood glucose levels are generally higher and less responsive to food intake than in mammals (Savory, 1987; Braun and Sweazea, 2008). Moreover, postprandial sampling time may also influence plasma glucose concentrations, which peak approximately 1 h after feeding and are no longer elevated 6 h postprandially in ducks (Tavernier et al., 2017b).

Finally, several limitations should be acknowledged, which also highlight potential avenues for improvement. First, neither individual nor group feeding behavior was monitored, preventing us from precisely characterizing the timing and duration of feeding within each one-hour meal. Second, the relatively high temperatures recorded during the feeding periods may have influenced feeding behavior and, consequently, feed intake. In addition, the diet used was the standard growing diet, which may have a lower capacity to promote hepatic lipid deposition than the starch-rich force-feeding diet. Finally, the relatively small sample sizes at some time points, combined with the assessment of gene expression at a single time point (6 hours after feeding) and the absence of corresponding protein-level measurements, may have reduced our ability to detect some potentially relevant effects.

## Conclusion

The fattening phase initially induced an increase in feed intake during the first day, accompanied by higher hepatic levels of total lipids and triglycerides, as well as an increase in hepatic glycogen content. These changes in liver composition were associated with an upregulation of genes involved in lipogenesis and glycogenesis. However, feed intake was rapidly regulated from the second day of the fattening phase onward, leading to a progressive decrease in liver weight as well as in hepatic total lipid and triglyceride content. In contrast, hepatic glycogen content remained at its maximal level throughout the entire fattening phase. Thus, this protocol did not induce a progressive accumulation of hepatic lipids across meals, primarily due to the transient nature of hyperphagia. While it remains far from constituting a viable alternative to conventional force-feeding practices, it nonetheless provides new insights into the regulation of food intake and the modulation of hepatic metabolism in a context of fractionated feeding. It also maintains the prospect of an alternative based entirely on voluntary feed intake, without the physical constraints associated with confinement or animal handling, which could represent a major improvement in animal welfare compared with force-feeding. Moreover, the observation of lipid storage during the initial days of the protocol highlights a rapid shift toward energy reserve deposition. This opens promising avenues for future research aimed at developing alternative feeding strategies to force-feeding, or at least potentially shortening the force-feeding period. The next challenge will be to move beyond this initial lipid storage phase in order to promote sustained lipid accumulation. This could be explored by testing different feed characteristics (e.g., palatability or lipogenic potential), extending the acclimation period, modifying light exposure, or combining these approaches with complementary strategies.

## Authors contribution

**Zwick LL**. contributed to

− Substantive input into the conception and design of the work, and the acquisition, analysis, and interpretation of data.
− Writing the manuscript draft and reviewing it critically for crucial intellectual content.
− Reviewing and explicitly approving the exact, final version of the manuscript before it goes to press.
− Agreeing to be personally and publicly responsible for all aspects of the work, ensuring that any questions regarding the accuracy or integrity of the research are thoroughly investigated and resolved.

**Szczepaniak R.** contributed to:

− Substantive input into the acquisition, analysis, and interpretation of data.
− Writing the manuscript draft and reviewing it critically for crucial intellectual content.
− Reviewing and explicitly approving the exact, final version of the manuscript before it goes to press.
− Agreeing to be personally and publicly responsible for all aspects of the work, ensuring that any questions regarding the accuracy or integrity of the research are thoroughly investigated and resolved.

**Coustaud K**. contributed to

− Substantive input into the conception and design of the work, and acquisition of data.
− Writing the manuscript draft and reviewing it critically for crucial intellectual content.
− Reviewing and explicitly approving the exact, final version of the manuscript before it goes to press.
− Agreeing to be personally and publicly responsible for all aspects of the work, ensuring that any questions regarding the accuracy or integrity of the research are thoroughly investigated and resolved.

**Galbadrakh T**. contributed to

− Substantive input into the acquisition, analysis, and interpretation of data.
− Writing the manuscript draft and reviewing it critically for crucial intellectual content.
− Reviewing and explicitly approving the exact, final version of the manuscript before it goes to press.
− Agreeing to be personally and publicly responsible for all aspects of the work, ensuring that any questions regarding the accuracy or integrity of the research are thoroughly investigated and resolved.

**Lasserre M**. contributed to:

− Substantive input into the conception and design of the work, and acquisition of data.
− Writing the manuscript draft and reviewing it critically for crucial intellectual content.
− Reviewing and explicitly approving the exact, final version of the manuscript before it goes to press.
− Agreeing to be personally and publicly responsible for all aspects of the work, ensuring that any questions regarding the accuracy or integrity of the research are thoroughly investigated and resolved.

**Fericelli Y**. contributed to:

− Substantive input into the acquisition, analysis, and interpretation of data.
− Writing the manuscript draft and reviewing it critically for crucial intellectual content.
− Reviewing and explicitly approving the exact, final version of the manuscript before it goes to press.
− Agreeing to be personally and publicly responsible for all aspects of the work, ensuring that any questions regarding the accuracy or integrity of the research are thoroughly investigated and resolved.

**Ricaud K.** contributed to:

− Substantive input into the acquisition, analysis, and interpretation of data.
− Writing the manuscript draft and reviewing it critically for crucial intellectual content.
− Reviewing and explicitly approving the exact, final version of the manuscript before it goes to press.
− Agreeing to be personally and publicly responsible for all aspects of the work, ensuring that any questions regarding the accuracy or integrity of the research are thoroughly investigated and resolved.

**Biasutti S.** contributed to:

− Substantive input into the acquisition, analysis, and interpretation of data.
− Writing the manuscript draft and reviewing it critically for crucial intellectual content.
− Reviewing and explicitly approving the exact, final version of the manuscript before it goes to press.
− Agreeing to be personally and publicly responsible for all aspects of the work, ensuring that any questions regarding the accuracy or integrity of the research are thoroughly investigated and resolved.

**Gontier K.** contributed to:

− Substantive input into the conception and design of the work, and acquisition, analysis, and interpretation of data.
− Writing the manuscript draft and reviewing it critically for crucial intellectual content.
− Reviewing and explicitly approving the exact, final version of the manuscript before it goes to press.
− Agreeing to be personally and publicly responsible for all aspects of the work, ensuring that any questions regarding the accuracy or integrity of the research are thoroughly investigated and resolved.

**Roy J.** contributed to:

− Substantive input into the conception and design of the work.
− Writing the manuscript draft and reviewing it critically for crucial intellectual content.
− Reviewing and explicitly approving the exact, final version of the manuscript before it goes to press.
− Agreeing to be personally and publicly responsible for all aspects of the work, ensuring that any questions regarding the accuracy or integrity of the research are thoroughly investigated and resolved.

**Panserat S.** contributed to:

− Substantive input into the conception and design of the work.
− Writing the manuscript draft and reviewing it critically for crucial intellectual content.
− Reviewing and explicitly approving the exact, final version of the manuscript before it goes to press.
− Agreeing to be personally and publicly responsible for all aspects of the work, ensuring that any questions regarding the accuracy or integrity of the research are thoroughly investigated and resolved.

**Houssier M.** contributed to:

− Substantive input into the conception and design of the work, and the acquisition, analysis, and interpretation of data.
− Writing the manuscript draft and reviewing it critically for crucial intellectual content.
− Reviewing and explicitly approving the exact, final version of the manuscript before it goes to press.
− Agreeing to be personally and publicly responsible for all aspects of the work, ensuring that any questions regarding the accuracy or integrity of the research are thoroughly investigated and resolved.

## Disclosures

The authors declare the following financial interests/personal relationships which may be considered as potential competing interests: HOUSSIER Marianne reports financial support was provided by Comité Interprofessionnel des Palmipèdes à Foie Gras (CIFOG). ZWICK Laura-Lou reports financial support was provided by Comité Départemental des Landes (CD40). ZWICK Laura-Lou reports financial support was provided by INRAE département PHASE. If there are other authors, they declare that they have no known competing financial interests or personal relationships that could have appeared to influence the work reported in this paper.
