# Supplementary material for: Adaptive responses to fractionated feeding: transient hyperphagia and reversible hepatic lipid deposition in mule ducks
Source: Poult Sci. 2026 Sep 16;105(12):107790. doi: 10.1016/j.psj.2026.107790 (PMC13625788; doi:10.1016/j.psj.2026.107790)
Supplement: Supplementary file 1 [file mmc1.docx]

**Supplemental Table 1. Detailed Composition of duckling starter feed (PAG1301, Nutricia, route de Saint Sever, Haut Mauco, BP27, France) and duck and goose growth feed (PAG1320, Nutricia, route de Saint Sever, Haut Mauco, BP27, France).**

| Category | Constituent / Additive | Starter Feed (PAG1301) | Growth Feed (PAG1320) |
| --- | --- | --- | --- |
| Analytical Constituents | Crude Protein | 17.50% | 15.00% |
|  | Crude Fat | 2.20% | 2.00% |
|  | Crude Fiber | 6.20% | 6.30% |
|  | Crude Ash | 6.20% | 5.20% |
|  | Lysine | 0.90% | 0.69% |
|  | Methionine | 0.33% | 0.29% |
|  | Calcium | 1.19% | 1.00% |
|  | Phosphorus | 0.61% | 0.50% |
|  | Sodium | 0.15% | 0.14% |
| Vitamins | Vitamin D3 (3a671) | 3000 IU/KG | 3000 IU/KG |
|  | Vitamin C (3a300) | 30 MG/KG |  |
|  | Vitamin A (3a672a) | 9000 IU/KG | 7000 IU/KG |
|  | Betaine Hydrochloride (3a925) | 50.1 MG/KG |  |
| Binders / Anti-Caking Agents | Bentonite (1m558i) | 0.02% |  |
|  | Sepiolite (1g562) | 63.0 MG/KG |  |
|  | Clinoptilolite (1g568) | 0.06% |  |
| Amino Acids | Hydroxyl Analog of Methionine | 0.15% | 0.10% |
| Digestibility Enhancers | 4a19 6-Phytase (EC 3.1.3.26) | 1500 FTU/KG | 1000 FTU/KG |
|  | Endo-1,4-β-xylanase (EC 3.2.1.8) | 1250.01 UV/KG | 150 FXU/KG |
|  | Endo-1,3(4)-β-glucanase (EC 3.2.1.6) | 860.01 UV/KG |  |
| Trace Elements | Copper (3b405 sulfate II pentahydrate) | 12 MG/KG | 15 MG/KG |
|  | Iron (3b103 sulfate (II) monohydrate) | 30 MG/KG | 24 MG/KG |
|  | Manganese (3b502 oxide) | 70 MG/KG | 50 MG/KG |
|  | Iodine (3b202 calcium iodate anhydrous) | 1.20 MG/KG | 1.20 MG/KG |
|  | Zinc (3b605 sulfate monohydrate) | 60 MG/KG | 35 MG/KG |
|  | Selenium (sodium selenite 3b801) | 0.30 MG/KG | 0.20 MG/KG |
| Main Ingredients | Wheat | - | - |
|  | Southwest Corn (Class A) | - | - |
|  | Sunflower Extraction Meal | - | - |
|  | Sorghum | - |  |
|  | Rapeseed Extraction Meal | - | - |
|  | Corn Distillers Grains with Solubles (DDGS) | - |  |
|  | Calcium Carbonate | - | - |
|  | Corn Gluten | - |  |
|  | Dicalcium Phosphate | - |  |
|  | Sodium Chloride | - | - |
|  | Boldo Processing Product | - |  |
|  | Yeast Products | - |  |
|  | Sorbitol | - |  |
|  | Wheat Bran | - | - |

**Supplemental Table 2. Nucleotide sequences of the PCR primers used to evaluate mRNA relative expressions of by real-time quantitative PCR.**

| Gene (name) | Gene (symbole) | Forward Primer Sequence | Reverse Primer Sequence |
| --- | --- | --- | --- |
| Enolase 1 | *ENO1* | CGCTACATGGGGAAAGGTGT | AAGGTCAGCAATGTGACGGT |
| Hexokinase 1 | *HK1* | GAGGGAGCAGACGTTGTGAA | GCCGCATCTCCTCCATGTAA |
| Pyruvate Dehydrogenase Alpha 1 | *PDHA1* | CTGCAGAGCTGGAAAAGGTC | CACTGCCACATCAATTTCCTT |
| Solute Carrier Family 2 Member 2 | *GLUT2* | GGAGTTGACCZZCCCGTTTA | CCCACCTCGAAGAAGATGAC |
| Glycogen Synthase 2 | *GYS2* | GTGCCACCATCACCTTCTGTC | TTCCTGTACTCTCCATGCTGCTTC |
| 1,4-Alpha-Glucan Branching Enzyme 1 | *GBE1* | TCACAGGTGACTTCAATGGCT | GCAGTTCGCCATTTTGAGCA |
| Glycogen Phosphorylase L | *PYGL* | CCGCGACTACTTCTTCGCCCT | GGTAGACGGCCTCGTCGCAG |
| ATP Citrate Lyase | *ACLY* | ACCCCACTGTTGGACTATGC | GCTTCAAGCGCTTCTGATCT |
| Acetyl-CoA Carboxylase | *ACC* | TGCCTCCGAGAACCCTAA | AAGACCACTGCCACTCCA |
| Fatty Acid Synthase | *FASN* | TGAAGAAGGTCTGGGTGGAG | CTCCAATAAGGTGCGGTGAT |
| Stearoyl-CoA Desaturase | *SCD1* | AGTGCTGCTCACATGTTTGG | TGAAGTCGATGAAGGCTGTG |
| ELOVL Fatty Acid Elongase 6 | *ELOVL6* | CTACTTTGTGCTCTTCTGCCATTTT | TGGCTTCTGTCTCATTTCTACCAC |
| Glycerol-3-Phosphate Acyltransferase, Mitochondrial | *GPAT1* | ACAACTTCAGCGGTCCTGTT | GCGCTGAGGTAGGAACGTAG |
| Choline/Ethanolamine Phosphotransferase 1 | *CEPT1* | CTGCTGTGCAGCTCTTTGAA | CGCAGTATCGAAGCAAATCA |
| Apolipoprotein B | *APOB* | TCTCACCGTGACTTGAGTGC | TCCCAGCAGAAGGTGAAGAT |
| Low-Density Lipoprotein Receptor | *LDLR* | TGTGGCCTTCAGAAAGCTCG | ATCTCGTGCTGCATGTAGGG |
| Microsomal Triglyceride Transfer Protein | *MTTP* | TGCAGATGGACAGAGTCGAG | GGATGCAGTGCTGAAAACCT |
| Acyl-CoA Oxidase 1 | *ACOX1* | CATGTTTGAGTGGGAAAGA | TTTTCAGGGCAGGAAAATTG |
| carnitine palmitoyltransferase 1A | *CPT1A* | GATTTGGACCAGTGGCTGAT | GAAGGTTGCTTTGCACCAAT |
| Acyl-CoA Synthetase Long Chain Family Member 1 | *ACSL1* | GGCTGGCTTCATACAGGAGA | CTCTTTTCTTGGCCCATTTG |
| Acyl-CoA Dehydrogenase Family Member 11 | *ACAD11* | TGGTTGTACCTCGAGCTGTG | CATCCACATGAGAGGGCTTT |
| Hepatic Triacylglycerol Lipase | *LIPC* | TCTGCCCATCACAGTTCTTG | TGTTTCCCCTGCTTTCACTC |
| Luciferase | *LUCIFERASE* | CATTCTTCGCCAAAAGCACTCTG | AGCCCATATCCTTGTCGTATCCC |
| Glyceraldehyde-3-Phosphate Dehydrogenase | *GAPDH* | CAGAGGACCAGGTTGTCTCC | CACCACACGGTTGCTGTATC |
| Stabilin 1 | *STAB1* | CCTTGGAAACCCACAGAAAA | TACAGTCACCGCTGCTGAAG |
